# Intracellular and Extracellular Platinum Quantification at Single-Cell Scale with LA-ICP-TOFMS

**DOI:** 10.1021/acsomega.6c03629

**Published:** 2026-07-10

**Authors:** Elisabeth Foels, Martin Schaier, Slavica Zdravac, Claude Molitor, Hoang Anh Nguyenová, David Loibnegger, Dina Baier-Romfeld, Walter Berger, Michael Jakupec, Gunda Koellensperger

**Affiliations:** † 27258 University of Vienna, Faculty of Chemistry, Institute of Analytical Chemistry, Waehringer Strasse 38, 1090 Vienna, Austria; ‡ 27258 University of Vienna, Vienna Doctoral School in Chemistry (DoSChem), Waehringer Strasse 42, 1090 Vienna, Austria; § 27258 University of Vienna, Faculty of Chemistry, Institute of Inorganic Chemistry, Waehringer Strasse 42, 1090 Vienna, Austria; ∥ Medical University of Vienna, Center for Cancer Research and Comprehensive Cancer Center, Borschkegasse 8a, 1090 Vienna, Austria

## Abstract

Understanding the localized fate of metal-based therapeutics is critical for optimizing anticancer efficacy and mitigating systemic toxicities, including nephrotoxicity, neurotoxicity, and ototoxicity. While platinum-based drugs are known to interact with both healthy and tumor cells as well as the extracellular matrix (ECM), conventional methods often lack the spatial resolution required to distinguish between these compartments. Here, we present an analytical framework integrating immunohistochemistry (IHC) with high-resolution laser ablation inductively coupled plasma mass spectrometry (LA-ICP-MS). While preserving spatial architecture, this approach enabled the partitioning of intracellular and extracellular platinum fractions in a three-dimensional multicellular tumor spheroid model (MDA-MB-468), revealing that 23% of the total platinum mass remained associated with the added collagen matrix. The robustness of this framework was further validated in murine spleen and kidney tissues. By applying area-based normalization to account for varying cell densities, we successfully differentiated distribution trends within complex regions, such as the splenic red and white pulp. This strategy provides a quantitative tool for assessing drug penetration and compartmentalized biodistribution in complex biological matrices.

## Graphical abstract

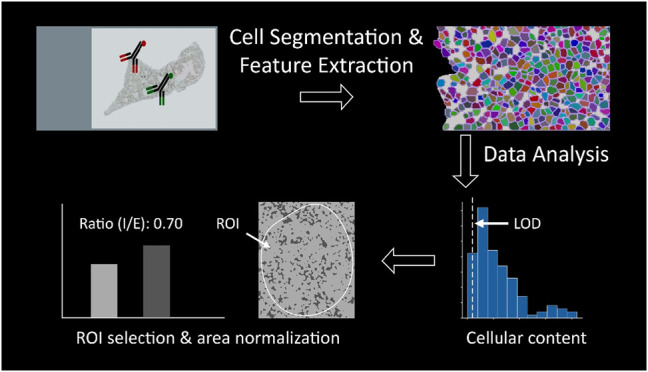

## Introduction

Ever since the serendipitous discovery of cisplatin, platinum-based anticancer drugs continue to be a central pillar of chemotherapy.^6–9^ While the clinically established platinum anticancer drugs are known to act by cross-linking to the cellular target DNA,^10–13^ for a plethora of candidate metal-based drugs the mode of action and targets are less defined, making the actual translation into clinical application complex.^14,15^

Today, it is well accepted that a cancer cell-centric view alone cannot unravel the multifaceted modes of action of metal compounds in anticancer therapy. These agents function *in vivo* within complex tissues, where the tumor microenvironment (TME)—a dynamic network of stromal and immune cells, vasculature, extracellular matrix (ECM), soluble mediators, and physicochemical gradients—governs drug access, activation, speciation, reactivity, and ultimately therapeutic outcome.^16–18^ Equally essential is the characterization of drug fate within normal, nonmalignant tissues, as comparing these structured environments to the tumor microenvironment reveals how biological architecture influences metal accumulation and retention.^19^ This shift in perspective has been accelerated by multiparametric single-cell technologies, such as mass cytometry and imaging mass cytometry, revealing how tissue heterogeneity shapes drug distribution and response.^19,20^ To accurately map these dynamics, it is essential to distinguish between intracellular metal loads, which have reached their primary targets, and the extracellular fraction, which remains associated with the structural ECM or the interstitial space. The ECM is a critical noncellular compartment of the TME, consisting of a complex network of proteins (*e.g.*, collagen, fibronectin, elastin, and laminin) that provide structural stability to complex tissues.^16–18^

Very early on in metal-based drug development, it was recognized that measuring intracellular drug accumulation supports smart design, as the intracellular accumulation was found to be a primary determinant of whether a metal-based anticancer drug will hit its intracellular target and/or overcome resistance.^21^ The extent of target modification and efficacy are tightly connected. ICP-MS and ICP-OES were indispensable measurement platforms for the field.^22^ With the introduction of ICP-MS based single-cell analysis, it became possible to associate cellular uptake with cell type, state and function at single-cell level overcoming the limits of bulk analysis.^23^ In fact, by averaging measurands for a cell population (bulk analysis), correlations found only for small subpopulations could be masked, even in “simple” *in vitro* model experiments.^24,25^ The first ICP-MS based single-cell studies addressing metal-based drugs were entirely method oriented. Omitting spatial analysis and in-depth cell phenotyping, these studies focused either on absolute metal drug quantification and or on correlations of platinum drug uptake with the cell cycle and hypoxia *in vitro* and *in vivo*.^26–28^

While elemental bioimaging by laser ablation ICP-MS has been broadly applied to screen tissue distribution and penetration of metal compounds at spatial resolution >1 μm, research integrating the toolbox of imaging mass cytometry (metal-labeled antibodies and single-cell resolution) is only emerging.^23,29^ Chang et al. used the principles of immunohistochemistry (IHC) and imaging mass cytometry (IMC) to study cisplatin distribution in tumor and normal tissues from cisplatin-treated mice bearing xenograft tumors, covering elements in a mass range of 75 to 209.^19^ The authors observed a widespread association of platinum with collagen fibers in both tumor and normal mouse tissues, indicating that not only the heterogeneous cell population, but also the extracellular matrix forming a dynamic structure around cells is essential for the drug supply and activity. Time-course studies showed slow release of stroma-bound platinum with unknown biological activity.^19^ Another study underscoring the role of the TME in platinum chemotherapy applied a combination of clinically established immunohistology with platinum bioimaging by LA-ICP-MS.^30^ Albeit no single-cell resolution was provided, platinum accumulation could still be clearly observed at the tissue architecture scale within cancer-associated fibroblast regions. Braun and Schaier et al. integrated imaging mass cytometry and quantitative bioimaging, creating a tool suitable for quantifying metals in single phenotypically characterized cells.^5^ It is the first pipeline allowing to report single-cell quantitative data retaining spatial information and multiparametric tissue characterization both at tissue architecture- and single-cell scale. Recently, the method was applied to an *in vivo* study on oxaliplatin showcasing the power of this integrated toolbox for spatial single-cell biology.^20^ Using xenograft models it could be shown that a cancer cell intrinsic resistance phenotype massively impacted on metal distribution dynamics in the cancer microenvironmental space.

In this work we expand the quantitative capabilities of our MeXpose approach, addressing intracellular *versus* extracellular metal-based drug accumulation in a spatial context. The approach goes beyond quantitative bioimaging, as it integrates single-cell segmentation.

Acknowledging that metal-based anticancer drugs are influenced to a large extent by extracellular chemistry and tissue architecture, because of their activity, speciation, and distribution, rather than by intracellular targets alone,^30^ we hypothesize that a method capable of quantifying cellular *versus* extracellular metal content in a tissue sample would substantially support drug development. Understanding the extent of metal drug load in the ECM matters because ECM-bound drug can act as a depot that sustains or spatially biases exposure, create pockets of high local reactivity (*e.g.*, increased adduct formation or ROS generation for redox-active complexes), and contribute to resistance by reducing intracellular delivery;^18^ conversely, excessive ECM accumulation may increase off-target toxicity (fibrosis, vascular damage) or impair immune cell function.^31^

We scrutinize the potential and limits of the proposed approach using an advanced *in vitro* three-dimensional (3D) multicellular tumor spheroid model and *in vivo* murine tissues, representing a progression toward higher structural complexity and cell density.

## Methods

### Chemicals and Reagents

For all dilutions and washing steps, ultrapure water (18.2 MΩ cm) was sourced from an ELGA Purelab Ultra MK2 system (High Wycombe, United Kingdom).

For the preparation of the standard solutions nitric acid (>69%, Rotipuran Supra) from Carl Roth (Karlsruhe, Germany) was used. The stock solutions of the standards were purchased from Labkings (Hilversum, The Netherlands). The fish gelatin, derived from cold-water fish skin, was purchased from Sigma-Aldrich (Vienna, Austria).

For the immunostaining tris buffered saline (BioUltra, pH 7.6), bovine serum albumin (lyophilized powder, BioReagent), m-Xylene (anhydrous, ≥99%), and ethanol (absolute, EMSURE), were purchased from Sigma-Aldrich (Vienna, Austria). The target retrieval solution (pH 9), containing Tris/EDTA, was obtained from Agilent Technologies (Waldbronn, Germany). Tween-20 detergent solution (stabilized as a 10% (w/v) solution) was bought from Thermo Fisher (Vienna, Austria). The Superblock blocking buffer was purchased from Fisher scientific (Vienna, Austria). The 16% paraformaldehyde (aqueous solution, EM grade) was purchased as glass ampules from Science Services (Munich, Germany). The fluorescent markers FITC-WGA (Wheat Germ Agglutinin) (5 mg/mL) and DAPI staining solution (1 μg/mL) were purchased from Szabo Scandic (Vienna, Austria). The obtained Anti-WGA from Szabo Scandic (Vienna, Austria) was labeled with the Maxpar X8 Antibody Labeling Kit (using 152Sm and 154Sm isotopes) from Standard BioTools (San Francisco, CA, USA), at a final concentration of 2 mg/mL.

Chemicals required for the cell culture experiments, including cell culture media and reagents, were purchased from Sigma-Aldrich (Vienna, Austria). The fetal calf serum (FCS) was obtained from PAA (Linz, Austria) and Serana (Brandenburg, Germany).

### External Calibration Standards

As external calibration standards, gelatin-based multielement microdroplet standards were used. The procedure for the preparation of the standards was published elsewhere.^1^

Briefly, a multielement calibration series was prepared gravimetrically. Consequently, commercially available standards were diluted with a one percent nitric acid solution and spiked with a gelatin stock solution to obtain a one percent gelatin solution. The calibration standards, including a blank solution, were transferred into a well plate and printed with a microdroplet generator (CellenONE X1, Cellenion, Lyon, France) onto glass slides. This results in droplet volumes of approximately 300–350 picolitre (pL) and droplet diameters of around 100–200 μm. Absolute amounts can be calculated and then used for quantification (Table S1).

### 3D Cell Culture

The MDA-MB-468 cell line was kindly provided by the University Hospital of the Medical University of Vienna (Vienna, Austria). The cells were cultured in StableCell Dulbecco’s Modified Eagle’s Medium/Ham’s Nutrient Mixture F12 (DMEM/F12, Sigma-Aldrich, Vienna, Austria) supplemented with 0.1% insulin (Sigma-Aldrich, Vienna, Austria) and 10% FCS (Mexican origin, Serana, Brandenburg, Germany).

The cells were seeded into 96-well plates (round-bottom, ultralow attachment, Corning) at 1.5 × 10^3^ cells per well, centrifuged at 1400 rpm for 3 min and then incubated overnight at 37 °C under 5% CO_2_. To support spheroid formation, collagen (1 μg/mL in DMEM/F12) (Collagen type I, rat tail, Corning) was added on the next day, followed by centrifugation at 900 rpm for 3 min. In total, the spheroids were grown in the incubator for 96 h from seeding until they were treated. Treatment was carried out with 10 μM oxaliplatin (BD146411, BLD Pharm; stock 0.5 mM in DMEM/F12) for 24 h. The spheroids were then embedded in Tissue-Tek O.C.T. Compound using Tissue-Tek cryomolds (both from Sakura). Sections of 5 μm thickness were prepared with a cryostat microtome (Leica CM3050 S).

### Cell Culture for Animal Experiments

The HCT116 human CRC cell line was kindly provided from Dr. Vogelstein of Johns Hopkins University (Baltimore, USA).^2^

The cells were cultured in McCoy′s medium (Sigma-Aldrich, Vienna Austria) supplemented with 10% fetal calf serum (PAA, Linz, Austria) and 2 mM glutamine (Sigma-Aldrich, Vienna, Austria). Cell culture experiments were conducted under standard conditions, including routine checks for Mycoplasma contamination.

### *In Vitro* Cell Viability Assay

To assess cell viability and the influence of oxaliplatin treatment on the cells, 3 × 10^3^ HCT116 cells were seeded in a 96-well plate, and 100 μL of McCoy’s medium was added to each well. The cells were allowed to recover for 24 h, after which oxaliplatin was prepared at the respective concentrations in additional 100 μL of medium. The cells were then continuously treated for 72 h. Cell viability was assessed using an MTT-based survival assay (EZ4U; Biomedica, Vienna, Austria).^3^

### Animal Experiment

For *in vivo* experiments, 1 × 10^6^ HCT116 cells suspended in serum-free RPMI medium (R6504, Sigma-Aldrich, St. Louis, MO, USA) were subcutaneously (s.c.) injected into the right flank of 11-week-old male CB-17/SCID mice. The experiments were conducted in accordance with the regulations of the Ethics Committee for the Care and Use of Laboratory Animals at the Medical University Vienna (proposal number BMWF-66.009/0140-II/3*b*/2011), the U.S. Public Health Service Policy on Human Care and Use of Laboratory Animals, as well as the United Kingdom Coordinating Committee on Cancer Prevention Research’s Guidelines for the Welfare of Animals in Experimental Neoplasia. The mice were housed in a pathogen-free environment within a laminar airflow cabinet. Animals were monitored daily for signs of distress. Tumour-bearing animals (*n* = 4) received intraperitoneal injections twice weekly for 2 weeks with either 9 mg/kg oxaliplatin (OxPt) or physiological saline containing 5% glucose as a solvent control. For imaging experiments, animals received a single intraperitoneal dose of oxaliplatin and were euthanized 72 h post-treatment. Organs were fixed in 4% formaldehyde solution (Carl Roth, Karlsruhe, Germany) for 24 h and then embedded in paraffin wax using a KOS machine (Milestone Medical, Sorisole, Italy).

### Multimodal Immunostaining of 3D Culture Cryosections

The cryosection (5 μm thickness) was fixed in 4% PFA in TBS for 30 min at 4 °C, followed by three washing steps in TBS/0.05% Tween, each for 5 min on an orbital shaker with gentle agitation. To block nonspecific binding sites, the section was incubated with Superblock buffer at room temperature for 30 min in a hydration chamber. Then, the section was incubated with 1:100 Fc Block in TBS/0.05% Tween for 10 min. For the metal-labeled antibody mix, the antibodies were diluted 1:50 in a solution containing 0.5% BSA and Fc Block (1:100) in TBS/0.05% Tween. The solution was centrifuged at 13,000*g* for 2 min to prevent the formation of aggregates. The section was then incubated with the antibody solution overnight at 4 °C. A detailed list of the antibodies used is provided in Table S2.

The following day, the section was first washed three times with TBS/0.05% Tween for 5 min each. It was then incubated with WGA (5 mg/mL), diluted 1:100 in TBS/0.05% Tween, for 10 min. The slide was subsequently washed with TBS/0.05% Tween for 5 min, followed by incubation with Iridium intercalator (125 μM, diluted 1:100) and DAPI (1:3) in TBS/0.05% Tween for 5 min in a hydration chamber. Finally, the slide was washed twice with TBS/0.05% Tween and once with water (each for 5 min), and air-dried.

### Multimodal Immunostaining of FFPE Murine Spleen and Kidney Sections

First, the FFPE sections (5 μm thickness) were baked in an oven for 1 h at 60 °C, followed by dewaxing in xylene for 20 min. Rehydration was performed using an ethanol gradient (100%, 95%, 80%, 70%) for 5 min at each concentration. Subsequently, the slides were washed in ultrapure water for a period of 5 min on an orbital shaker with gentle agitation. Heat-induced antigen retrieval was carried out at 96 °C for 30 min, followed by careful cooling to room temperature. The slides were then washed with ultrapure water and TBS/0.05% Tween for 10 min each. Followed by the incubation with the preincubated FITC-WGA/Anti-WGA (1 μL WGA (5 mg/mL) and 3 μL Anti-WGA (2 mg/mL) in 96 μL TBS/0.05% Tween) for 30 min in a hydration chamber filled with TBS. The preincubation of the FITC-labeled WGA and metal-labeled WGA were carried out for 1 h at room temperature. To block nonspecific binding sites, the sections were incubated with SuperBlock buffer for 30 min in a hydration chamber. The tissues were then incubated in a 1:100 dilution of CD16/CD32 prepared in TBS/0.05% Tween for 10 min in a hydration chamber. The metal-labeled antibody mixtures (1:50 dilution) were prepared in a solution containing 0.5% BSA and 1:100 CD16/CD32 in TBS/0.05% Tween. To prevent aggregate formation, the antibody solutions were centrifuged at 13,000*g* for 2 min. The tissue sections were incubated with the antibody cocktail overnight at 4 °C in a hydration chamber. Details of the antibodies are listed in Tables S3 and S4.

The sections were then washed three times with TBS/0.05% Tween, followed by staining with an Iridium intercalator (125 μM, diluted 1:100) and the addition of DAPI (1:3) in TBS/0.05% Tween for 5 min in a hydration chamber. Finally, the slides were washed twice with TBS/0.05% Tween (5 min each), once with ultrapure water (5 min), and air-dried.

### LA-ICP-TOFMS Analysis

The LA-ICP-TOFMS measurements were performed with an Iridia 193 nm laser system from Teledyne Photon Machines (Bozeman, MT, USA), equipped with the low dispersion ablation cell in the Cobalt ablation chamber. The laser system was connected *via* the aerosol rapid introduction system (ARIS) to the icpTOF 2R from Tofwerk AG (Thun, Switzerland).

Before entering the plasma, an argon (Ar) makeup gas (0.95–1.00 mL/min) was introduced into the helium (He) carrier gas (0.60 mL/min) in the mixing bulb of the ARIS. The instrument parameters were daily tuned while ablating NIST SRM612 certified reference material from the National Institute for Standards and Technology (Gaithersburg, MD, USA). The instrumental parameters were optimized based on the intensities for ^59^Co^+^, ^115^In^+^ and ^238^U^+^, and low oxide formation (^238^U^16^O^+^/^238^U^+^ ratio <3%), as well as low elemental fractionation (^238^U^+^/^232^Th^+^ ratio ∼ 1).

All laser measurements were conducted at a repetition rate of 500 Hz. For the spleen measurements, circular spot sizes of 3 and 2 μm were used, with fixed dosage mode 3 and 4, and a *y*-direction interspacing between the lines of 1 μm, 750 or 500 nm, resulting in a pixel size of 1 μm, 750 and 500 nm, respectively. The kidney data were acquired using a circular spot size of 2 μm, with a fixed dosage mode of 4 and a *y*-direction interspacing of 500 nm, yielding in a pixel size of 500 nm. For the spheroid measurement, a circular spot size of 3 μm was used, with a fixed dosage mode 3, and a *y*-direction interspacing between the lines of 1 μm, resulting in a pixel size of 1 μm. The laser energy densities used for ablating the standards and samples were chosen to exceed the ablation threshold of the gelatin standards and biological material while remaining below that of the glass substrate. It should be noted that, for the small spot sizes utilized, the actual fluence at the sample surface is lower than the set values, primarily due to diffraction effects. Additionally, the actual fluence may vary depending on laser calibration and optical condition.

The icpTOF 2R ICP-TOFMS allows the measurement of a broad mass range from *m*/*z* 14 to 256, with a specified mass resolution (*R* = *m*/Δ*m*) of 6000, defined at full width at half-maximum (FWHM). All measurements were performed in collision cell technology (CCT) mode, in which the collision/reaction cell is pressurized with a H_2_/He gas mixture. The gas flow rate was set to 4.20 mL/min to minimize interferences in the low mass range. A detailed list of instrumental parameters is provided in Table S5.

### Data Acquisition and Processing

Data recording was conducted using TofPilot (version 2.15.11, Tofwerk AG, Thun, Switzerland), with raw data stored in the HDF5 (open-source hierarchical data format). Further processing was performed in HDIP (version 1.8.5.171, Teledyne Photon Machines, Bozeman, MT, USA), where an integrated script automatically converted the HDF5 files into 2D elemental distribution maps. These data sets were exported as raw TIFF files for single-cell evaluation.

### Calibration and Quantification

Quantification was performed by selecting regions of interest (ROIs) for the gelatin standards within HDIP. The summed relative elemental signal intensities were correlated with the known absolute amounts of the standards to construct linear calibration curves. The limit of detection (LOD) was determined using pixel-based data from the multielement standards, following previously established procedures.^4^

### Computational Analysis and Single-Cell Segmentation

Data evaluation was performed using the MeXpose data analysis pipeline.^5^ This pipeline integrates Cellpose (versions 3.1.1.1 and 4.0.6) for cell segmentation and CellProfiler (version 4.2.4) for feature extraction. Initial statistical analysis, including cellular quantification and phenotyping, followed the established version 1.0 framework, while an updated version 2.0 script was employed for advanced population gating, and regional subanalysis (code can be accessed at https://github.com/koellenspergerlab/mexpose).

Cell segmentation for the spheroid and spleen sections was performed using fluorescence-based structural markers. Nuclei were stained with DAPI, and cell boundaries were identified using WGA (wheat germ agglutinin) staining, enabling delineation of cellular regions. For the kidney section, a metal-labeled anti-WGA antibody (^154^Sm) and an iridium-based DNA intercalator were used to enable segmentation of cellular structures. The resulting segmentation masks were subsequently applied to the aligned LA-ICP-TOFMS images to extract intracellular and extracellular platinum-associated signals.

### Intracellular and Extracellular Signal Quantification

As an extension of the MeXpose_v2 single-cell analysis module, we developed an interactive tool to extract spatial signal distributions within user-defined (regions of interest) ROIs. The method classifies pixels as intracellular (mask value >0) or extracellular (mask value = 0) based on Cellpose segmentation masks, with ROI polygons converted to binary pixel masks using scikit-image (v0.24.0). Cells at ROI boundaries are included if their centroids fall within the selection, though only pixels strictly within the ROI contribute to the statistics. Intracellular regions are defined by the Cellpose-derived segmentation masks. The extracellular compartment is defined as the complementary image area within the ROI not assigned to the intracellular mask. Because erythrocytes could not be reliably segmented as a distinct compartment in complex tissue using the applied marker panel and a nucleus-based segmentation approach, erythrocyte-associated platinum was assigned to the extracellular fraction. Within this computational framework, the extracellular compartment therefore represents all noncell-associated regions/signals within the selected ROI and may include matrix-associated and vascular spaces depending on the tissue architecture. For each ROI, summed signal intensities and pixel counts are computed separately for intracellular and extracellular compartments. The tool was implemented in Python (v3.12) using NumPy (v2.1.3), Pandas (v2.2.3), Plotly (v5.24.1), Matplotlib (v3.9.2), Pillow (v11.0.0), imageio (v2.36.0), SciPy (v1.14.1), and ipywidgets (v8.1.5), with analyses performed without any additional normalization steps. The code used for these analyses is available in the notebooks/extensions/intra_extra directory of the MeXpose repository (https://github.com/koellenspergerlab/mexpose).

These summed intensities were subsequently quantified using external calibration, converting raw intensities into absolute amounts expressed in femtograms (fg). To account for morphological heterogeneity across different tissue regions (selected ROIs), these values were normalized to the corresponding pixel area of each compartment, yielding area-normalized platinum levels in fg/μm^2^.

### Fluorescence Imaging

Immunofluorescence (IF) imaging was conducted on a Zeiss Axioscope 7 microscope, equipped with an Axiocam 305 mono camera and a Colibri 7 LED illumination system.

## Results and Discussion

### Framework for Single-Cell and Compartmental Platinum Quantification

Three-dimensional (3D) cell culture models better replicate spatial organization than 2D monolayers, facilitating relevant investigations of drug uptake and signal transduction.^32^ Incorporating matrices such as collagen gels into 3D models, or utilizing multicellular spheroids that actively secrete collagen and fibronectin, creates a valuable system for studying cells and the extracellular matrix (ECM) *in vitro* before transitioning to more complex *in vivo* studies.^33,34^ This is critical, as the ECM is a fundamental component of the tumor microenvironment, directly modulating nutrient supply and drug penetration.^35^

To reliably map the distribution of metal-based drugs within dense biological matrices and to distinguish between intra- and extracellular platinum (Pt) levels, specific analytical criteria must be met. High-resolution imaging (≤1 μm) is essential to resolve fine structural features. Integrating immunohistochemistry (IHC) with metal-labeled antibodies (*e.g.*, collagen type I) allows the ECM to be differentiated from cellular components. Targeted membrane and nuclei markers then provide the foundation for precise cell segmentation. This is particularly critical in models with high cell density, where dense biological architectures necessitate robust spatial definitions to ensure accurate compartmental distribution analysis. While segmentation parameters are precisely optimized, achieving absolute accuracy remains an inherent challenge in high-throughput tissue imaging, especially in dense sections. However, the use of deep-learning models such as Cellpose, which provides quality control through visualization and the ability to interactively correct segmentation errors, ensures that the resulting quantitative trends remain representative. To move from qualitative imaging toward a quantitative assessment, a calibration strategy (*e.g.*, gelatin microdroplet standards) is essential. The MeXpose data analysis pipeline enables phenotypic characterization and quantification of metal content at the single-cell level within tissue sections.^5^ By converting intensity data into absolute amounts, it allows visualization of metal levels across cell populations and subpopulations, revealing quantitative differences between cellular compartments. Such information is essential to scrutinize the distribution of metal-based anticancer agents within complex tissue sections, determining whether they reach the target site and to which extent. However, as with all FFPE-based elemental imaging workflows, potential preparation-induced redistribution effects should be considered when interpreting absolute metal levels and compartment-specific ratios.

As an extension of the single-cell analysis module in MeXpose, an interactive visualization and analysis tool was developed. Within user-defined regions of interest (ROIs) intra- and extracellular compartments can be selectively extracted for further analysis, such as quantification and area-normalization. Resolving spatial variations is critical, as shown in 3D penetration studies.^36,37^ Specifically, Gutierrez-Romero et al. identified localized “hotspots” of cisplatin and Pt­(IV)-loaded nanoparticles in outer spheroid layers.^37^ Our approach advances this by further partitioning signals into intracellular and extracellular fractions. This distinction allows for the determination whether these “hotspots” reflect cellular internalization or extracellular accumulation. Consequently, this strategy provides the framework for the spatially resolved quantification of metal-based drugs and their localized fate within the biological matrices presented in the following sections.

To illustrate the enhancement of our strategy, Figure 1 provides a schematic overview of established approaches and our extension. Figure 1A shows the Pt distribution within a tissue section, where raw intensities are converted into absolute amounts *via* external calibration. While pixel-based quantification provides an overview of total Pt distribution, it lacks the detail to distinguish whether the metal is localized within cells or the surrounding matrix. The MeXpose pipeline (Figure 1B) enables quantitative cellular metal analysis by combining cell segmentation with external calibration, followed by single-cell analysis, gating, and limit of detection (LOD) determination. To extend these capabilities, the new module (Figure 1C) enables the partitioning of intra- and extracellular sections across the entire section or within user-defined regions of interest (ROIs). The exported data includes the pixel count of the intra- and extracellular regions, which can be converted into an area based on the selected pixel size. This enables area-normalization to account for morphological heterogeneity and facilitate quantitative comparisons between distinct ROIs. By normalizing to the specific area of each compartment, the framework accounts for both local cell density and variable ROI dimensions, ensuring a direct and unbiased comparison of drug accumulation across diverse tissue architectures.

**Figure 1:**
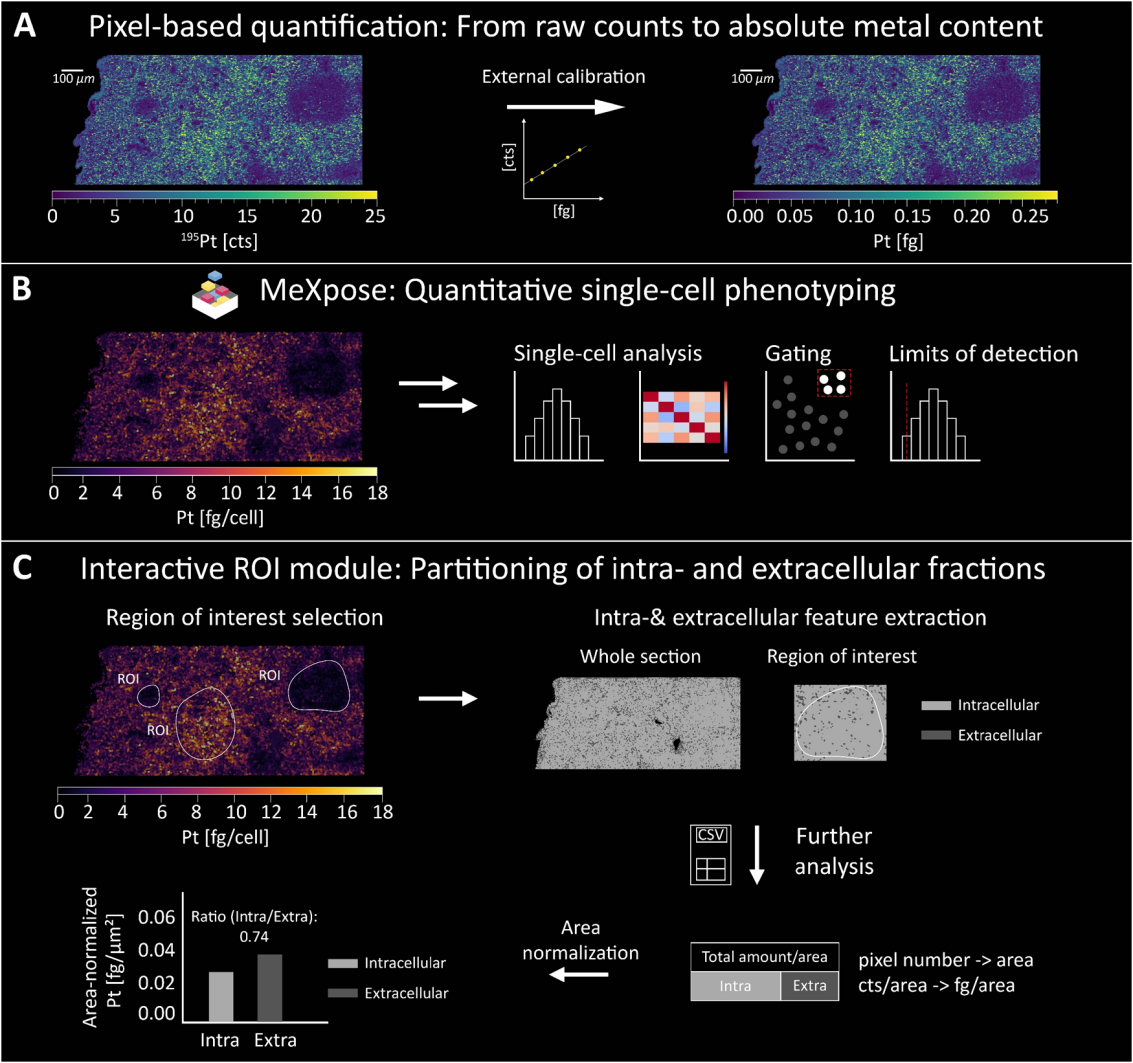
(A) Pixel-based quantification, where raw counts are converted into absolute amounts (fg, femtogram) using an external calibration. (B) The MeXpose data analysis pipeline, where quantitative cellular levels can be further analyzed, including single-cell analysis, gating, and the determination of the limit of detection (LOD). (C) The extension of the MeXpose pipeline, which enables the partitioning of intracellular and extracellular metal fractions. The module allows feature extraction across the entire tissue section or user-defined regions of interest (ROIs). Following data export, additional analysis can be performed, such as area normalization (fg/μm^2^), to enable spatially resolved quantitative comparisons of compartmentalized metal distribution.

To demonstrate the feasibility of this extension, we present two representative biological applications: 3D multicellular tumor spheroids and complex murine splenic and renal tissues.

### Spatially Resolved Platinum and DNA Damage in a 3D Cell Culture Model

In the 3D cell culture model, the extracellular environment comprised a collagen matrix integrated with MDA-MB-468 cells. Derived from a human breast tumor of epithelial origin, the MDA-MB-468 cell line exhibited a distinct distribution of pan-keratin, consistent with the formation of epithelial intermediate filaments (Figure S1). Collagen type I immunostaining confirmed the structural integrity of the extracellular space, while the presence of E-cadherin demonstrated that cell–cell adhesion was maintained upon oxaliplatin (OxPt) treatment (Figure S1).

The quantitative Pt distribution in the OxPt-treated 3D model is shown in Figure S2. To partition these signals into intra- and extracellular compartments, we integrated high-resolution elemental mapping with IHC-based cell segmentation, resolving 128 individual cells. The resulting spatial distribution and population heterogeneity are shown in Figure 2. The mean Pt content was 5.8 ± 4.5 fg/cell (mean diameter 9.7 ± 2.2 μm), significantly exceeding the 0.045 fg/cell LOD. Pearson correlation analysis revealed a moderate relationship between cell size and intracellular Pt levels (*R* = 0.65), suggesting that while cell size influences the absolute Pt load, drug distribution is also modulated by spatial and biological factors beyond physical cell volume. Intracellular Pt accounted for 77% (740 fg) of the total mass (958 fg), while the extracellular fraction represented 23% (218 fg). This distribution indicates that, while the drug successfully enters the cells, a significant portion remains associated with the collagen matrix, which may limit local bioavailability and modulate therapeutic efficacy.

**Figure 2:**
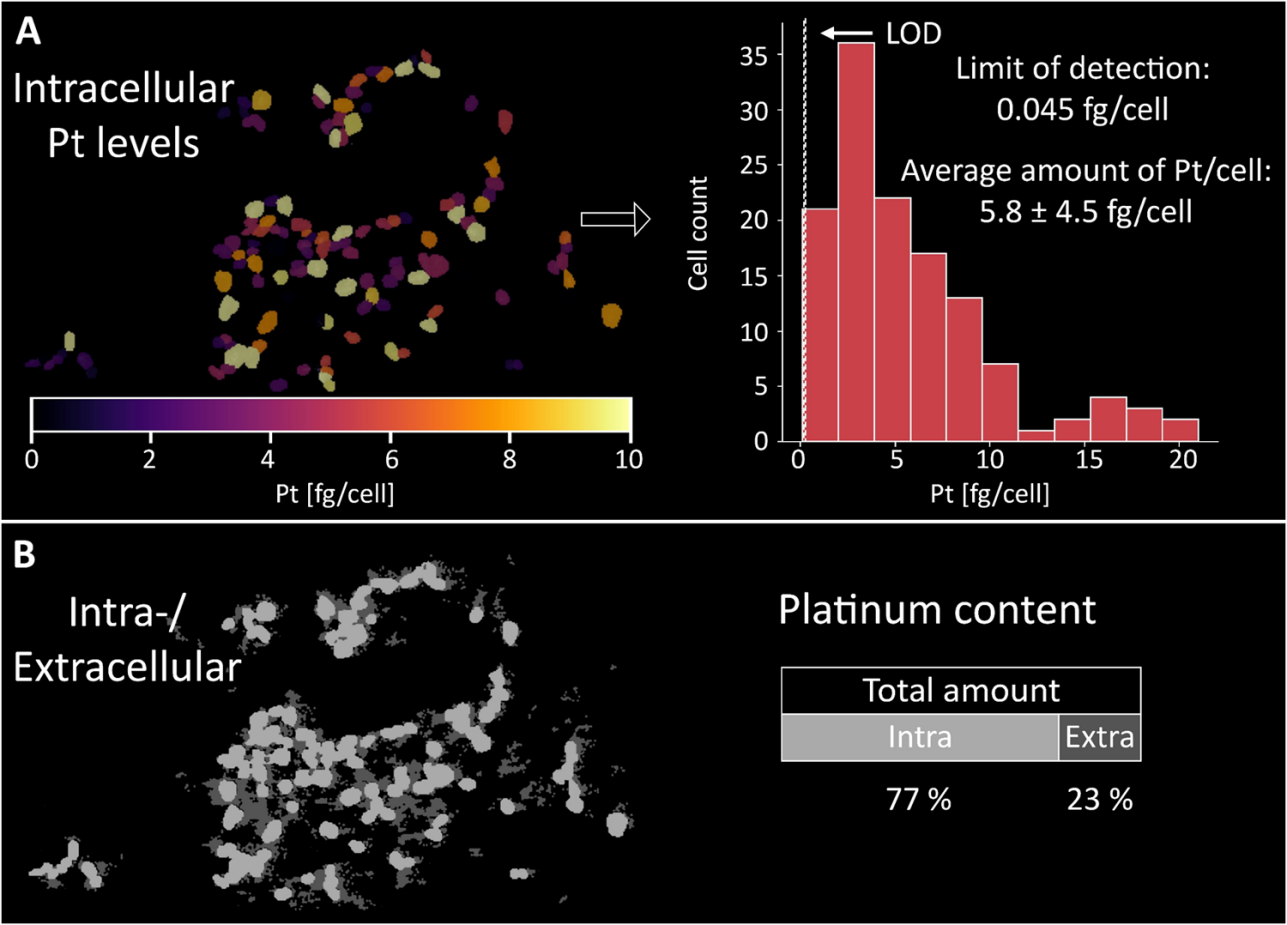
(A) The quantitative Pt distribution was assessed for 128 segmented cells. The histogram shows the distribution of Pt content per cell. Based on the gelatin microdroplet standards, a LOD for Pt of 0.045 fg/cell was determined. (B) The segmentation mask was used to analyze the intra- and extracellular Pt distribution within the 3D cell culture model. The bar plot illustrates the distribution of Pt between the intra- and extracellular regions. LA-ICP-TOFMS images were acquired with a pixel size of 1 μm at a repetition rate of 500 Hz.

To correlate these levels with biological response, DNA damage was assessed *via* pH2AX expression. Localized pH2AX hotspots were observed (Figure S1), likely reflecting a combination of OxPt-induced damage and physiological stress from 3D spheroid formation. To characterize this subpopulation, a manual gating strategy was employed to correlate pH2AX intensities with intracellular Pt levels (Figure 3A). This identified 35 cells with elevated damage, which exhibited a wide distribution of Pt levels (Figure 3B). In contrast, the remaining 93 cells (*n* = 93) showed baseline pH2AX levels despite similar Pt exposure (Figure 3C). This comparative analysis enables the direct correlation of Pt accumulation with the resulting biological response at the single-cell level.

**Figure 3:**
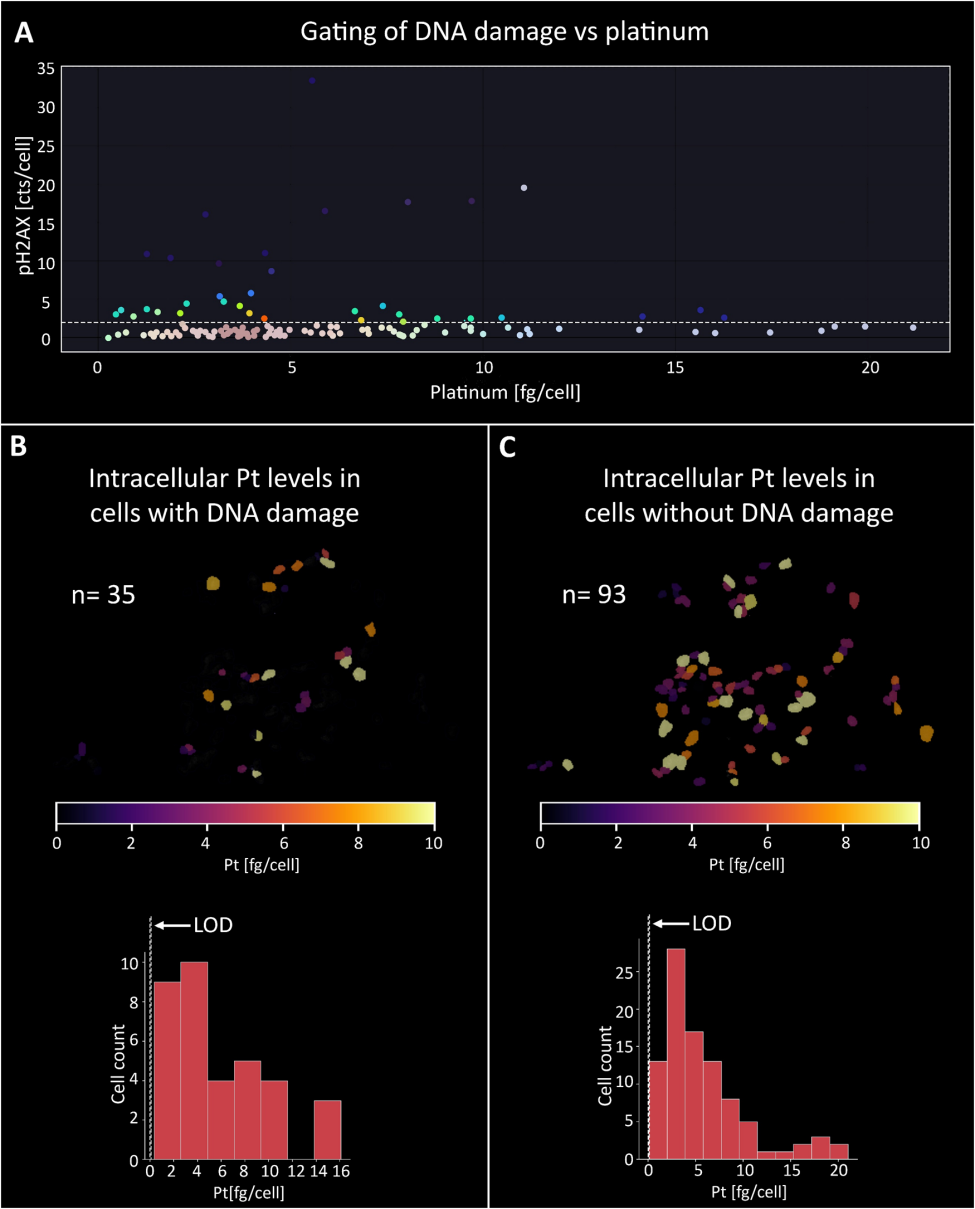
(A) Scatter plot representing the correlation between pH2AX and Pt levels upon OxPt treatment. The manual gating threshold was established to distinguish cell populations based on the pH2AX signal. (B) shows the spatial and quantitative Pt distribution within the subpopulation exhibiting elevated pH2AX expression, while (C) shows the Pt distribution for the cell population not associated with significant DNA damage.

### Compartmentalized Platinum Distribution in Splenic and Renal Tissues

Transitioning from a 3D cell culture model to mouse tissue introduces additional complexity due to the increased cellular heterogeneity and distinct extracellular compartments. Kidney and spleen sections from an OxPt-treated mouse were used to evaluate the methodology’s ability to differentiate compartmental Pt levels within more complex tissue architectures. Both organs exhibit well-defined extracellular regions for quantitative evaluation, with the spleen representing a particularly complex model due to its high cellular density.

The spleen consists of two main functional compartments: the red pulp and the white pulp. The red pulp facilitates blood filtration, marked by high endogenous iron levels from the removal of aged red blood cells. In contrast, the white pulp is densely populated with lymphocytes and immune surveillance. The iron distribution in Figure 4A clearly shows these regions, while nuclei and membrane marker (Figure 4B) reveal the higher cellular density of the white pulp. To characterize the tissue state, the proliferation marker Ki-67 and pH2AX were utilized (Figure S3), revealing ongoing cellular proliferation and increased DNA damage. Structural architecture was further defined by α-smooth muscle actin (α-SMA) and collagen type I to identify the capsule and trabeculae. These connective tissue structures, composed primarily of collagen and elastin, provide a foundation for investigating intra- and extracellular drug distributions (Figures 4A and S4). A similar approach was applied to the structural organization of the kidney. The renal cortex is covered by the capsule, a connective tissue layer primarily composed of collagen and elastic fibers (Figure S5). The inner medulla is predominantly cellular, supported by a specialized extracellular matrix essential for tissue organization. Figure S5 illustrates the endogenous iron distribution and the cellular arrangement within the cortical region and capsule. Both tissues possess distinct extracellular matrices, providing the necessary basis for compartmental differentiation.

**Figure 4:**
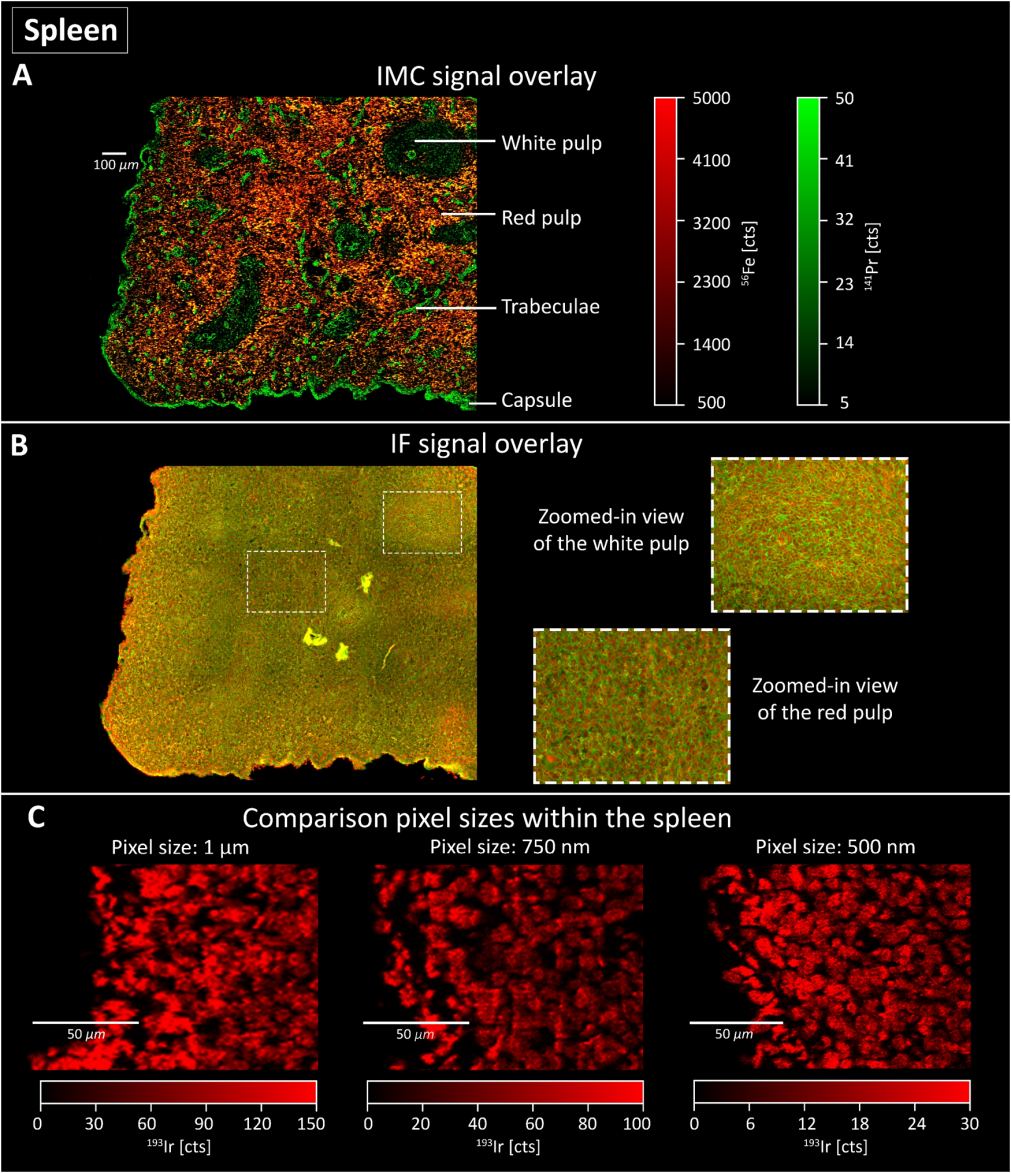
(A) shows an IMC signal overlay of iron (^56^Fe) and α-SMA (^141^Pr) in a spleen tissue section. The images were acquired at a pixel size of 1 μm and a repetition rate of 500 Hz, revealing distinct structural features in the spleen from an OxPt-treated mouse. The fluorescence overlay of DAPI and WGA in (B) provides an overview of the cellular structure. (C) presents iridium intensity maps at different pixel sizes: 1 μm, 750 nm, and 500 nm.

The ability to distinguish these regions strongly depends on the selected spatial resolution. To evaluate this effect, various pixel sizes were used to analyze different spleen regions (Figures 4C and S6). While higher spatial resolution is critical for single-cell analysis and fine detail, it inherently reduces signal intensity. A balance must therefore be maintained between maximizing structural detail and ensuring sufficient signal for robust cell segmentation and statistical analysis.

To investigate intra- and extracellular Pt distributions in greater detail, LA-ICP-TOFMS data were acquired at high spatial resolutions (1 μm for the spleen; 500 nm for the kidney). Overlays of the Pt and collagen signals for both tissue sections confirmed the presence of Pt within the extracellular matrix (Figure S4).

Segmentation of the spleen section resolved 25917 individual cells (Figure S7). Figure 5A illustrates the quantitative Pt distribution across these cells, with zoomed-in regions highlighting elevated Pt within the red pulp compared to the lower levels within the white pulp. This distribution pattern is likely driven by blood circulation; the red pulp, being highly vascularized for blood filtration, experiences greater exposure to circulating OxPt than the densely packed white pulp. Figure 5B presents the corresponding histograms for the segmented cells, showing a mean Pt content of 4.7 ± 3.1 fg/cell, which significantly exceeds the 0.046 fg/cell LOD determined by external calibration (Figure S8). The cellular diameter histogram reflects the diversity of splenic cell types, with a mean diameter of 8.7 ± 1.8 μm.

**Figure 5:**
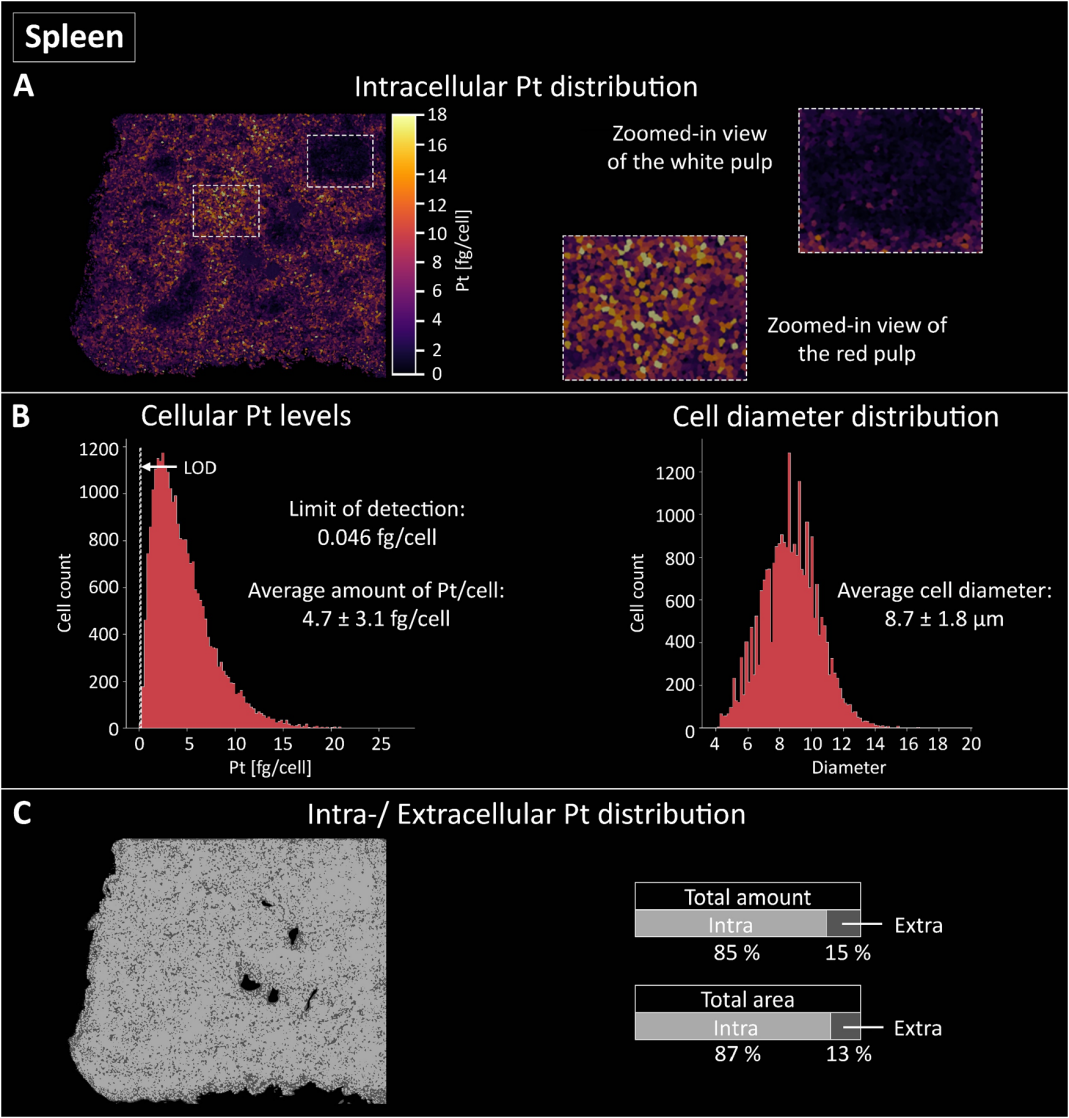
Data for the analysis of intra- and extracellular regions in a spleen tissue section were acquired using LA-ICP-TOFMS at a repetition rate of 500 Hz with a pixel size of 1 μm. Segmentation identified 25,917 cells in the spleen data set. (A) illustrates the cellular Pt distribution. (B) presents a histogram of Pt content per cell (4.7 ± 3.1 fg/cell). Based on the gelatin microdroplet standards, a LOD for Pt of 0.046 fg/cell was determined. The average cell diameter was 8.7 ± 1.8 μm. (C) depicts the intra- and extracellular regions of the tissue section. Cells located at the image borders, where only partial cell profiles were visible, as well as regions affected by tissue folding, were excluded from subsequent data analysis. The bar plots summarize the Pt distribution in the intra- and extracellular regions and their corresponding areas.

Using the segmentation mask, the tissue section was divided into intra- and extracellular compartments (Figure 5C). A quantitative comparison relative to area, revealed that approximately 85% of the total Pt was localized within intracellular regions, which occupied 87% of the total section area. These results emphasize the high cellular density of the spleen, where the majority of the tissue is occupied by cellular mass. The extracellular compartment accounted for 15% of the Pt and 13% of the area.

To gain a more detailed insight into Pt distribution, specific regions of interest (ROIs) within the spleen were analyzed (Figure 6A). Statistical analysis (Figure 6B) confirmed significantly higher Pt levels within red pulp cells (*n* = 2178) compared to white pulp cells (*n* = 2113), supporting the initial observations in Figure 5A.

**Figure 6:**
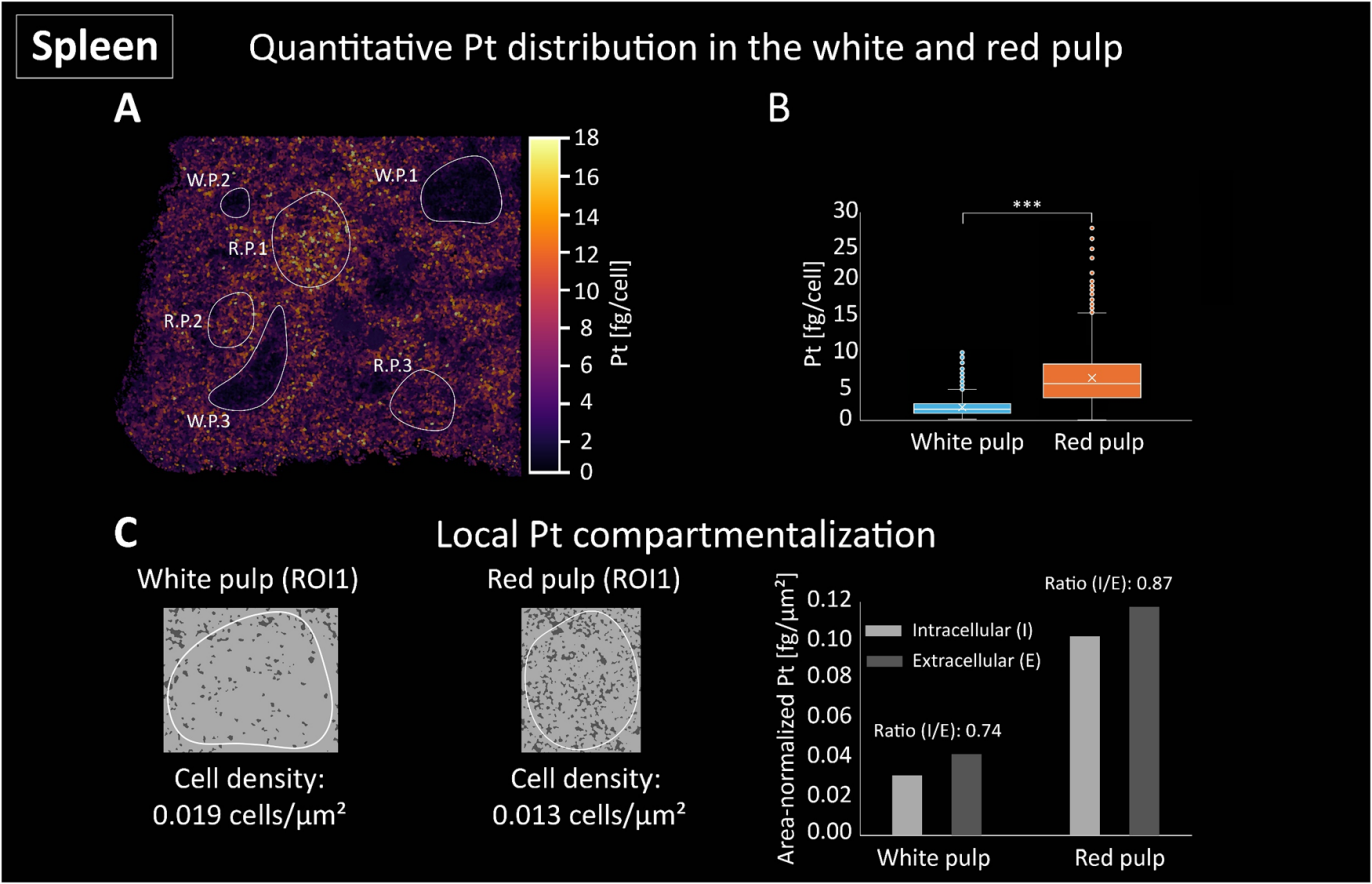
(A) LA-ICP-TOFMS imaging of a spleen tissue section with spatial resolution of 1 μm; the map displays cellular Pt levels in fg/cell. Various regions of interest (ROIs) for the white pulp (W.P.) and red pulp (R.P.) were selected for comparative analysis. (B) statistical comparison confirms significantly higher Pt levels per cell in the red pulp (*n* = 2113) compared to the white pulp (*n* = 2178), determined using Welch’s *t* test (*** *p* < 0.001). (C) shows detailed compartmentalization of Pt in selected ROIs. The area-normalized Pt content [fg/μm^2^] reveals that both ROIs exhibit higher relative Pt levels in the extracellular space, with intra/extracellular ratios of 0.74 (white pulp) and 0.87 (red pulp), respectively.

Analysis of these ROIs (Figure 6C) revealed distinct differences in cellular arrangement and compartmental distribution. While the white pulp exhibited a slightly higher cell density (0.019 cells/μm^2^) compared to the red pulp (0.013 cells/μm^2^), the area-normalized Pt content was found to be higher in the red pulp. In both regions, Pt levels were higher in the extracellular space than in the intracellular compartment. Specifically, the extracellular space showed a 26% higher Pt accumulation in the white pulp (I/E ratio: 0.74) and a 13% higher accumulation in the red pulp (I/E ratio: 0.87). Detailed data for the remaining ROIs are provided in Tables S6 and S7.

For the kidney, a targeted analysis yielded 5076 segmented cells (Figure S7). Unlike the clear differentiation observed between the red and white pulp of the spleen (Figure 5A), the quantitative Pt distribution in the kidney did not exhibit distinct regions of enrichment (Figure 7A). The corresponding histogram in Figure 7B showed a mean Pt content of 1.5 ± 1.0 fg/cell, exceeding the 0.12 fg/cell LOD. This higher LOD compared to the spleen is primarily attributed to the smaller pixel size (500 nm), which reduces the S/N ratio per area. The mean cellular diameter was 9.2 ± 1.9 μm (Figure 7B).

**Figure 7:**
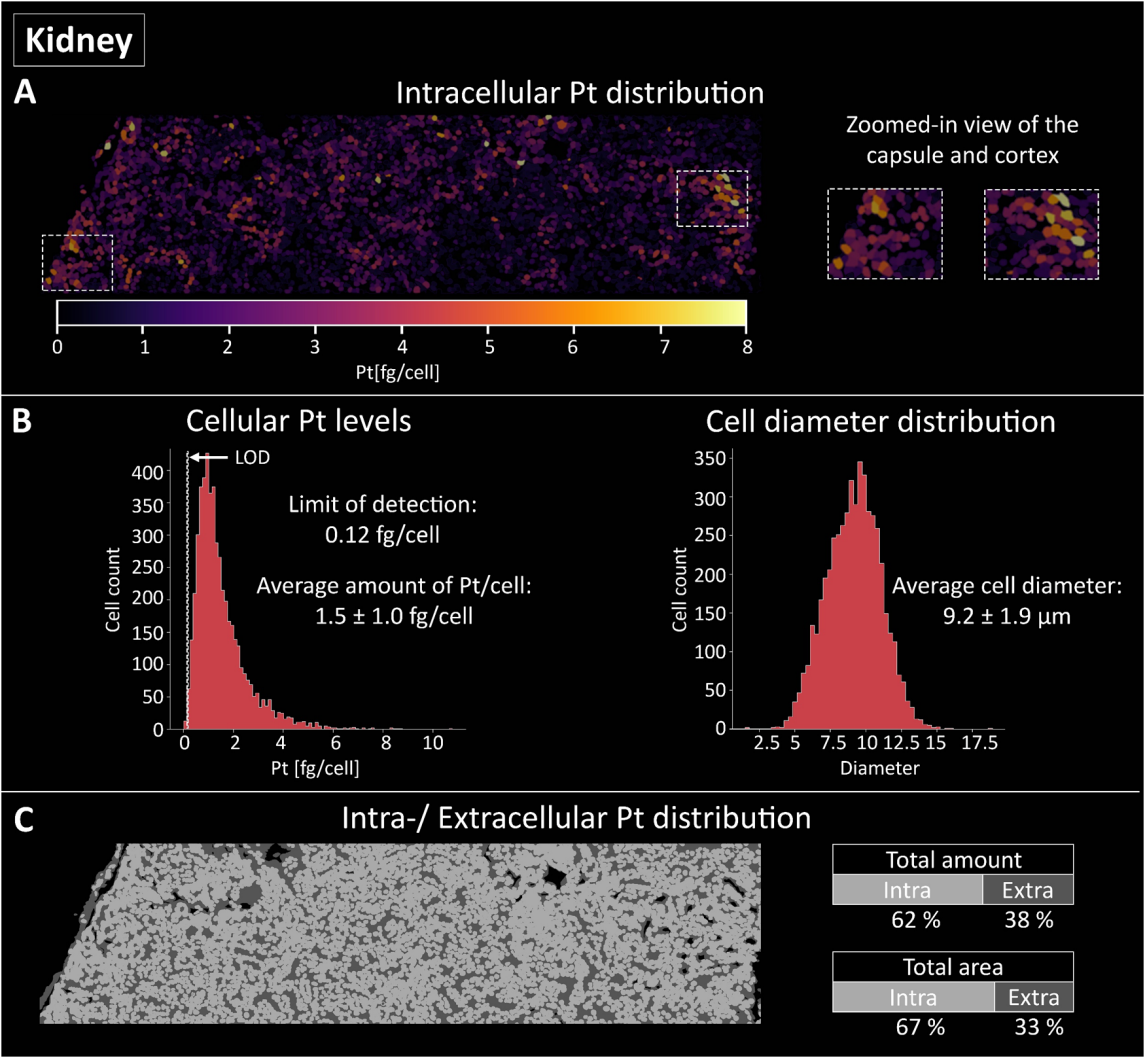
Data for the analysis of intra- and extracellular regions in a kidney tissue section were acquired using LA-ICP-TOFMS at a repetition rate of 500 Hz with a pixel size of 500 nm. Segmentation identified 5076 cells in the kidney data set. (A) illustrates the cellular Pt distribution. (B) presents a histogram of Pt content per cell (1.5 ± 1.0 fg/cell). Based on the gelatin microdroplet standards, a LOD for Pt of 0.12 fg/cell was determined. The average cell diameter was 9.2 ± 1.9 μm. (C) depicts the intra- and extracellular regions of the tissue section. Cells located at the image borders, where only partial cell profiles were visible, as well as regions affected by tissue folding, were excluded from subsequent data analysis. The bar plots summarize the Pt distribution in the intra- and extracellular regions and their corresponding areas.

Partitioning the tissue *via* a segmentation mask revealed that approximately 62% of the Pt was localized within intracellular regions, covering 67% of the tissue area. The extracellular compartment accounted for 38% of the Pt and 33% of the area (Figure 7C). While the kidney serves as the primary route for OxPt elimination, the accumulation levels observed here likely reflect the transient passage of the drug during excretion. This suggests that even at lower cellular concentrations compared to highly perfused organs like the spleen, persistent renal exposure during the elimination process remains a factor in potential nephrotoxicity.

## Conclusions

In this study, we present an analytical framework for the spatially resolved quantification of metal-based drugs across both a 3D cell culture model and murine tissue sections. By integrating high-resolution LA-ICP-TOFMS with immunohistochemistry and advanced image analysis tools, we successfully partitioned platinum signals into intra- and extracellular fractions at the single-cell scale.

Our findings demonstrate that oxaliplatin (OxPt) is distributed across both compartments in all investigated models. In the 3D spheroid model, while the drug was effectively internalized by cells, a significant portion (23%) remained associated with the added collagen matrix. Extending this workflow to complex splenic and renal tissues further demonstrated its applicability in high-density cellular environments. The ability to extract ROI-specific, area-normalized platinum levels provided critical insights into distribution patterns, such as elevated accumulation in the splenic red pulp compared to the white pulp. Notably, within both splenic compartments, a higher relative accumulation of platinum was observed in the extracellular space that would otherwise be obscured by varying cell densities and ROI dimensions.

Ultimately, this methodology provides a valuable tool for drug penetration studies, enabling a deeper understanding of how the extracellular microenvironment modulates drug bioavailability and localized therapeutic response. This workflow is applicable to a broad range of biological matrices, from *in vitro* 3D cell culture models to complex tissue sections. Furthermore, this approach is not limited to platinum-based compounds and can be readily adapted to investigate the biodistribution and efficacy of a wide range of metal-based therapeutics and diagnostics in anticancer research. The integration of additional structural markers enables future refinement of compartment definitions and expanded interrogation of tissue microenvironments. However, potential redistribution or partial loss of metal species during FFPE processing and staining procedures should be considered when interpreting absolute metal levels and compartment-specific ratios. Such effects may influence the apparent distribution between intracellular and extracellular fractions depending on the chemical form and tissue binding characteristics of the investigated species. Nevertheless, the primary aim of this study was to establish a proof-of-principle workflow for spatially distinguishing intra- and extracellular platinum distributions within complex biological matrices. Future studies directly comparing FFPE and snap-frozen sections will be valuable for assessing preparation-induced redistribution effects on absolute quantification.

## Supplementary Material



## Notes

External calibration standard composition; antibody panels for 3D spheroid, spleen, and kidney analyses; instrumental parameters; supplementary characterization and imaging data for spheroid, spleen, and kidney tissues; segmentation validation; platinum quantification workflows; and quantitative ROI analyses of splenic white and red pulp regions (PDF)

## References

[6] Rosenberg B., Van Camp L., Krigas T. (1965). Inhibition of Cell Division in *Escherichia coli* by Electrolysis Products from a
Platinum Electrode. Nature.

[7] Rosenberg B., Vancamp L., Trosko J. E., Mansour V. H. (1969). Platinum Compounds:
a New Class of Potent Antitumour Agents. Nature.

[8] Rosenberg B., VanCamp L. (1970). The Successful Regression
of Large Solid Sarcoma 180
Tumors by Platinum Compounds1. Cancer Res..

[9] Wheate N. J., Walker S., Craig G. E., Oun R. (2010). The status of platinum
anticancer drugs in the clinic and in clinical trials. Dalton Trans..

[10] Takahara P. M., Rosenzweig A. C., Frederick C. A., Lippard S. J. (1995). Crystal structure
of double-stranded DNA containing the major adduct of the anticancer
drug cisplatin. Nature.

[11] Jamieson E. R., Lippard S. J. (1999). Structure, Recognition,
and Processing of Cisplatin–DNA
Adducts. Chem. Rev..

[12] Chválová K., Brabec V., Kaspárková J. (2007). Mechanism of the formation
of DNA-protein cross-links by antitumor cisplatin. Nucleic Acids Res..

[13] Dilruba S., Kalayda G. V. (2016). Platinum-based drugs: past, present and future. Cancer Chemother. Pharmacol..

[14] Meier-Menches S. M., Gerner C., Berger W., Hartinger C. G., Keppler B. K. (2018). Structure–activity relationships
for ruthenium
and osmium anticancer agents – towards clinical development. Chem. Soc. Rev..

[15] Antonarakis E. S., Emadi A. (2010). Ruthenium-based chemotherapeutics: are they ready for prime time?. Cancer Chemother. Pharmacol..

[16] Chen S.-H., Chang J.-Y. (2019). New Insights into Mechanisms of Cisplatin
Resistance:
From Tumor Cell to Microenvironment. Int. J.
Mol. Sci..

[17] Mehraj U., Ganai R. A., Macha M. A., Hamid A., Zargar M. A., Bhat A. A., Nasser M. W., Haris M., Batra S. K., Alshehri B., Al-Baradie R. S., Mir M. A., Wani N. A. (2021). The tumor
microenvironment as driver of stemness and therapeutic resistance
in breast cancer: New challenges and therapeutic opportunities. Cell Oncol..

[18] Meng L., Zheng Y., Liu H., Fan D. (2024). The tumor
microenvironment:
a key player in multidrug resistance in cancer. Oncologie.

[19] Chang Q., Ornatsky O. I., Siddiqui I., Straus R., Baranov V. I., Hedley D. W. (2016). Biodistribution of cisplatin revealed by imaging mass
cytometry identifies extensive collagen binding in tumor and normal
tissues. Sci. Rep..

[20] Schaier M., Baier D., Theiner S., Berger W., Koellensperger G. (2025). LA-ICP-TOFMS
Imaging Reveals Significant Influence of Cancer Cell Resistance on
Oxaliplatin Compartmentalization in the Tumor Microenvironment. JACS Au.

[21] Hartinger C. G., Groessl M., Meier S. M., Casini A., Dyson P. J. (2013). Application
of mass spectrometric techniques to delineate the modes-of-action
of anticancer metallodrugs. Chem. Soc. Rev..

[22] Brouwers E. E. M., Tibben M., Rosing H., Schellens J. H. M., Beijnen J. H. (2008). The application of inductively coupled
plasma mass
spectrometry in clinical pharmacological oncology research. Mass Spectrom. Rev..

[23] Theiner S., Schoeberl A., Schweikert A., Keppler B. K., Koellensperger G. (2021). Mass spectrometry
techniques for imaging and detection of metallodrugs. Curr. Opin. Chem. Biol..

[24] Corte-Rodríguez M., Espina M., Sierra L. M., Blanco E., Ames T., Montes-Bayón M., Sanz-Medel A. (2015). Quantitative evaluation of cellular
uptake, DNA incorporation and adduct formation in cisplatin sensitive
and resistant cell lines: Comparison of different Pt-containing drugs. Biochem. Pharmacol..

[25] Qin Z., Ren G., Yuan J., Chen H., Lu Y., Li N., Zhang Y., Chen X., Zhao D. (2020). Systemic Evaluation
on the Pharmacokinetics of Platinum-Based Anticancer Drugs From Animal
to Cell Level: Based on Total Platinum and Intact Drugs. Front. Pharmacol..

[26] Chang Q., Ornatsky O. I., Koch C. J., Chaudary N., Marie-Egyptienne D. T., Hill R. P., Tanner S. D., Hedley D. W. (2015). Single-cell
measurement
of the uptake, intratumoral distribution and cell cycle effects of
cisplatin using mass cytometry. Int. J. Cancer.

[27] Corte
Rodríguez M., Álvarez-Fernández García R., Blanco E., Bettmer J., Montes-Bayón M. (2017). Quantitative
Evaluation of Cisplatin Uptake in Sensitive and Resistant Individual
Cells by Single-Cell ICP-MS (SC-ICP-MS). Anal.
Chem..

[28] Schoeberl A., Gutmann M., Theiner S., Schaier M., Schweikert A., Berger W., Koellensperger G. (2021). Cisplatin Uptake in Macrophage Subtypes
at the Single-Cell Level by LA-ICP-TOFMS Imaging. Anal. Chem..

[29] Chang Q., Ornatsky O. I., Siddiqui I., Loboda A., Baranov V. I., Hedley D. W. (2017). Imaging Mass Cytometry. Cytometry
Part A.

[30] Linares J., Sallent-Aragay A., Badia-Ramentol J., Recort-Bascuas A., Méndez A., Manero-Rupérez N., Re D. L., Rivas E. I., Guiu M., Zwick M., Iglesias M., Martinez-Ciarpaglini C., Tarazona N., Varese M., Hernando-Momblona X., Cañellas-Socias A., Orrillo M., Garrido M., Saoudi N., Elez E., Navarro P., Tabernero J., Gomis R. R., Batlle E., Pisonero J., Cervantes A., Montagut C., Calon A. (2023). Long-term platinum-based drug accumulation
in cancer-associated fibroblasts promotes colorectal cancer progression
and resistance to therapy. Nat. Commun..

[31] Hauge A., Rofstad E. K. (2020). Antifibrotic therapy
to normalize the tumor microenvironment. J.
Transl. Med..

[1] Schweikert A., Theiner S., Wernitznig D., Schoeberl A., Schaier M., Neumayer S., Keppler B. K., Koellensperger G. (2022). Micro-droplet-based
calibration for quantitative elemental bioimaging by LA-ICPMS. Anal. Bioanal. Chem..

[2] Jungwirth U., Xanthos D. N., Gojo J., Bytzek A. K., Körner W., Heffeter P., Abramkin S. A., Jakupec M. A., Hartinger C. G., Windberger U., Galanski M., Keppler B. K., Berger W. (2012). Anticancer
Activity of Methyl-Substituted Oxaliplatin Analogs. Mol. Pharmacol..

[3] Englinger B., Kallus S., Senkiv J., Heilos D., Gabler L., van Schoonhoven S., Terenzi A., Moser P., Pirker C., Timelthaler G., Jäger W., Kowol C. R., Heffeter P., Grusch M., Berger W. (2017). Intrinsic fluorescence of the clinically
approved multikinase inhibitor nintedanib reveals lysosomal sequestration
as resistance mechanism in FGFR-driven lung cancer. J. Exp. Clin. Cancer Res..

[4] Foels E., Braun G., Molitor C., Schaier M., Loibnegger D., Hendriks L., Koellensperger G. (2026). Evaluating
limits of detection for
single-cell elemental bioimaging by LA-ICP-TOFMS. Talanta.

[5] Braun G., Schaier M., Werner P., Theiner S., Zanghellini J., Wisgrill L., Fyhrquist N., Koellensperger G. (2024). MeXpose—A
Modular Imaging Pipeline for the Quantitative Assessment of Cellular
Metal Bioaccumulation. JACS Au.

[32] Van
Zundert I., Fortuni B., Rocha S. (2020). From 2D to 3D Cancer
Cell Models—The Enigmas of Drug Delivery Research. Nanomaterials.

[33] Sapudom J., Pompe T. (2018). Biomimetic tumor microenvironments
based on collagen matrices. Biomater. Sci..

[34] Arakawa A., Jakubowski N., Koellensperger G., Theiner S., Schweikert A., Flemig S., Iwahata D., Traub H., Hirata T. (2019). Imaging of
Ag NP transport through collagen-rich microstructures in fibroblast
multicellular spheroids by high-resolution laser ablation inductively
coupled plasma time-of-flight mass spectrometry. Analyst.

[35] Holle A. W., Young J. L., Spatz J. P. (2016). *In vitro* cancer
cell–ECM interactions inform *in vivo* cancer
treatment. Adv. Drug Delivery Rev..

[36] Theiner S., Van Malderen S. J. M., Van Acker T., Legin A., Keppler B. K., Vanhaecke F., Koellensperger G. (2017). Fast High-Resolution Laser Ablation-Inductively
Coupled Plasma Mass Spectrometry Imaging of the Distribution of Platinum-Based
Anticancer Compounds in Multicellular Tumor Spheroids. Anal. Chem..

[37] Gutierrez-Romero L., Gallego B., Blanco E., Karst U., Rodríguez R., Montes-Bayon M. (2025). Single cell combined with laser ablation
ICP-MS to
study cisplatinum (IV) loaded nanoparticles penetration pathways in
osteosarcoma spheroids. Anal. Chim. Acta.

