# Supplementary material for: Intracellular and Extracellular Platinum Quantification at Single-Cell Scale with LA-ICP-TOFMS
Source: ACS Omega. 2026 Jul 10;11(29):43908–20. doi: 10.1021/acsomega.6c03629 (PMC13425759; doi:10.1021/acsomega.6c03629)
Supplement: Supplementary file 1 [file ao6c03629_si_001.pdf]

Supplementary information

Intracellular and Extracellular Platinum Quantification at Single-Cell Scale with LA-ICP-TOFMS

Elisabeth Foels[1,2], Martin Schaier[1], Slavica Zdravac[2,3,] Claude Molitor[1,2,3], Hoang Anh Nguyenová[1,2], David Loibnegger[1,2,3], Dina Baier-Romfeld[3], Walter Berger[4], Michael Jakupec[3], Gunda Koellensperger[1]*

[1] University of Vienna, Faculty of Chemistry, Institute of Analytical Chemistry, Waehringer Strasse 38, 1090 Vienna, Austria

[2] University of Vienna, Vienna Doctoral School in Chemistry (DoSChem), Waehringer Strasse 42, 1090 Vienna, Austria

[3] University of Vienna, Faculty of Chemistry, Institute of Inorganic Chemistry, Waehringer Strasse 42, 1090 Vienna, Austria

[4] Medical University of Vienna, Center for Cancer Research and Comprehensive Cancer Center, Borschkegasse 8a, 1090 Vienna, Austria

* Corresponding author:

Gunda Koellensperger

Institute of Analytical Chemistry, Waehringer Strasse 38, 1090 Vienna, Austria

Tel: +43-1-4277-52303, Email: gunda.koellensperger@univie.ac.at

Table S1: List of elements included in the external calibration and their theoretical amounts in femtograms [fg].

Element	Blank [fg]	Standard 1 [fg]	Standard 2 [fg]	Standard 3 [fg]	Standard 4 [fg]	Standard 5 [fg]
Ag	0	170	343	681	1733	3467
Al	0	170	343	681	1733	3467
As	0	170	343	681	1733	3467
B	0	170	343	681	1733	3467
Ba	0	170	343	681	1733	3467
Be	0	170	343	681	1733	3467
Ca	0	170	343	681	1733	3467
Cd	0	170	343	681	1733	3467
Co	0	170	343	681	1733	3467
Cr	0	170	343	681	1733	3467
Cu	0	170	343	681	1733	3467
Fe	0	170	343	681	1733	3467
K	0	1700	3433	6811	17328	34667
Mg	0	170	343	681	1733	3467
Mn	0	170	343	681	1733	3467
Mo	0	170	343	681	1733	3467
Na	0	170	343	681	1733	3467
Ni	0	170	343	681	1733	3467
Pb	0	170	343	681	1733	3467
Sb	0	170	343	681	1733	3467
Se	0	170	343	681	1733	3467
Si	0	85	172	341	866	1733
Ti	0	170	343	681	1733	3467
Tl	0	170	343	681	1733	3467
V	0	170	343	681	1733	3467
Zn	0	170	343	681	1733	3467
Ru	0	166	335	665	1692	3353
Pt	0	167	337	668	1700	3500

Table S2: List of metal-labelled antibodies for 3D cell culture model.

Antibody target	Clone	Metal tag	Catalog number	Target
CD44	IM7	^{153}Eu	3153029D	Tumor/EMT
Ki-67	B56	^{168}Er	3168022D	Cell proliferation
pH2AX	N1-431	^{165}Ho	3165036D	DNA damage
Collagen Type I	Polyclonal	^{169}Tm	3169023D	Extracellular matrix
Pan-Keratin	C11	^{148}Nd	3148020D	Epithelial cells
E-Cadherin	24E10	^{158}Gd	3158029D	Epithelial cell membrane

Table S3: List of metal-labelled antibodies for spleen.

Antibody target	Clone	Metal tag	Catalog number	Target
CD44	IM7	^{153}Eu	3153029D	Tumor/EMT
Ki-67	B56	^{168}Er	3168022D	Cell proliferation
pH2AX	N1-431	^{165}Ho	3165036D	DNA damage
Collagen Type I	Polyclonal	^{169}Tm	3169023D	Extracellular matrix
α-SMA	1A4	^{141}Pr	3141017D	Smooth muscle cells
Anti-WGA		^{152}Sm		

Table S4: List of metal-labelled antibodies for kidney.

Antibody target	Clone	Metal tag	Catalog number	Target
CD44	IM7	^{153}Eu	3153029D	Tumor/EMT
Ki-67	B56	^{168}Er	3168022D	Cell proliferation
pH2AX	N1-431	^{165}Ho	3165036D	DNA damage
Collagen Type I	Polyclonal	^{169}Tm	3169023D	Extracellular matrix
α-SMA	1A4	^{141}Pr	3141017D	Smooth muscle cells
Anti-WGA		^{154}Sm		

Table S5: Instrument parameters.

**Laser ablation**	
Spot size [µm]	2-3
Interspacing [µm]	1-0.50
Dosage	3-4
Pixel size [µm]	1-0.50
Fluence [J cm^{-2}]	0.6-1.6
Repetition rate [Hz]	500
**ICP-TOFMS**	
Plasma Power [W]	1440
Sampling depth [mm]	2.7
Cone materials	Ni
Plasma gas flow [L min^{-1}]	14.0
Auxiliary gas flow [L min^{-1}]	0.80
Nebulizer gas flow [L min^{-1}]	0.95-1.00
Helium carrier gas flow[L min^{-1}]	0.60
CCT gas	93% He (v/v), 7% H_2 (v/v)
CCT gas flow [ml min^{-1}]	4.20
Mass range [m/z]	14-256

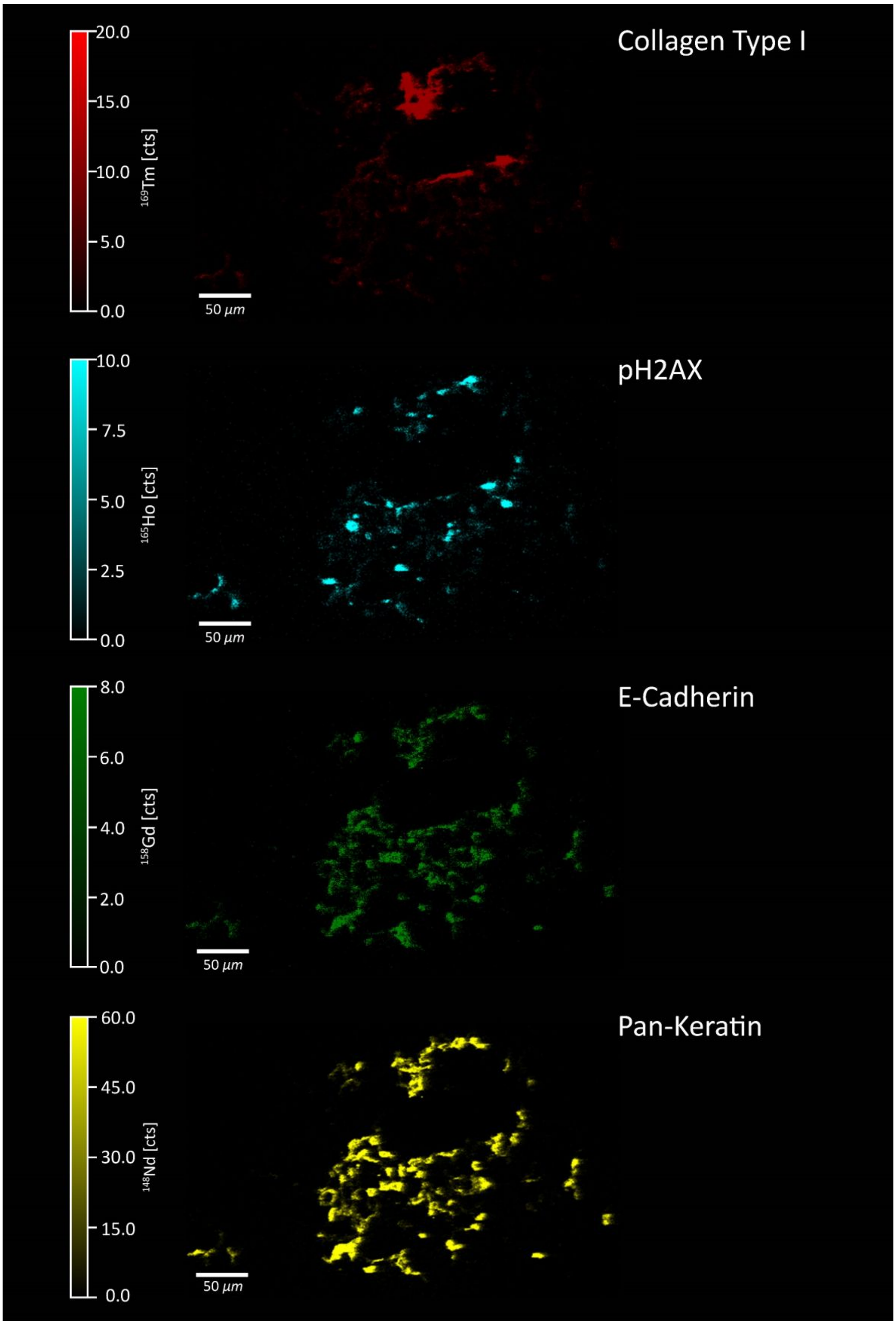

Figure S1: Characterization of the 3D multicellular tumour spheroid model. Visualization of collagen distribution, DNA damage and epithelial identity using Collagen Type I, pH2AX, E-Cadherin, and Pan-Keratin. LA-ICP-TOFMS data were acquired at 500 Hz with a pixel size of 1 µm.

Platinum quantification was performed at the pixel level using gelatine micro-droplet standards. Figure S2 illustrates the spatial distribution of the platinum signal across the 3D multicellular tumour spheroid model, where counts were converted into absolute amounts (femtograms) via external calibration.

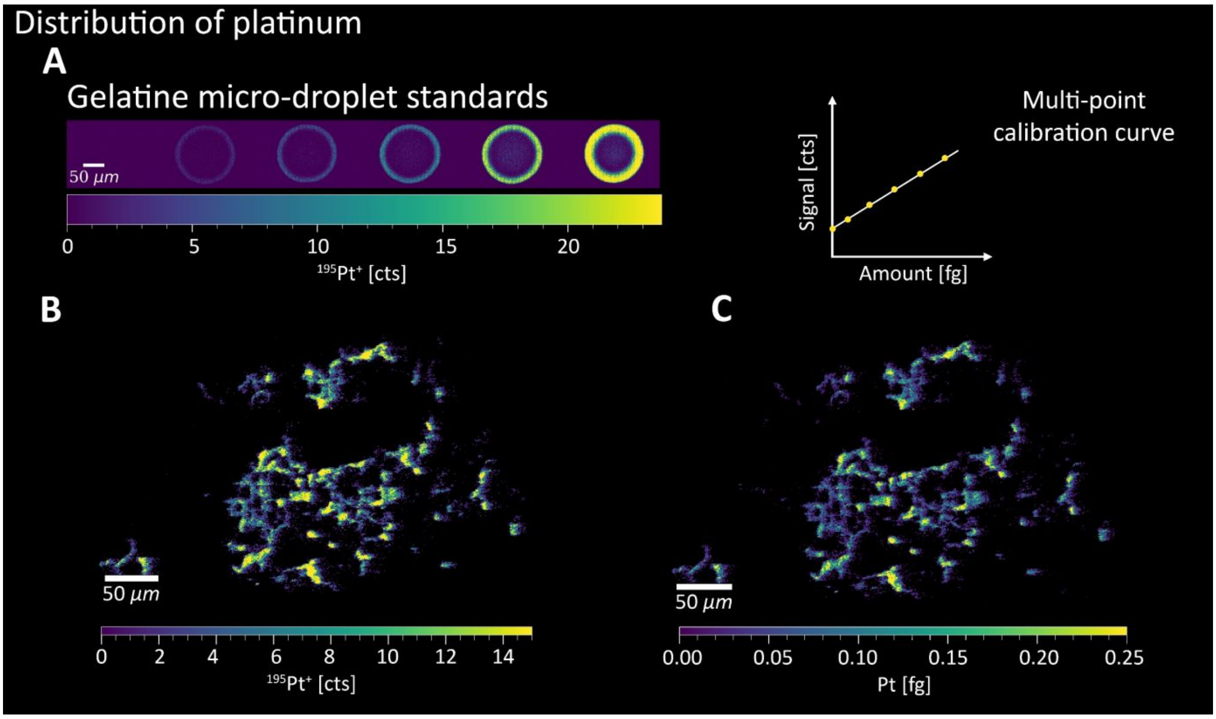

Figure S2: (A) illustrates a quantification series of gelatine micro-droplet standards, resulting in a multi-point calibration series, as shown in the schematic illustration. External calibration was used to convert platinum counts into absolute amounts. The absolute amount, given in femtograms, was obtained by dividing the counts by the slope of the external calibration curve. In (B), the platinum distribution of the 3D multicellular tumour spheroid model is shown in counts, and in (C), the corresponding platinum levels are converted into absolute amounts. LA-ICP-TOFMS images of the 3D multicellular tumour spheroid model were acquired with a pixel size of 1 µm at a repetition rate of 500 Hz.

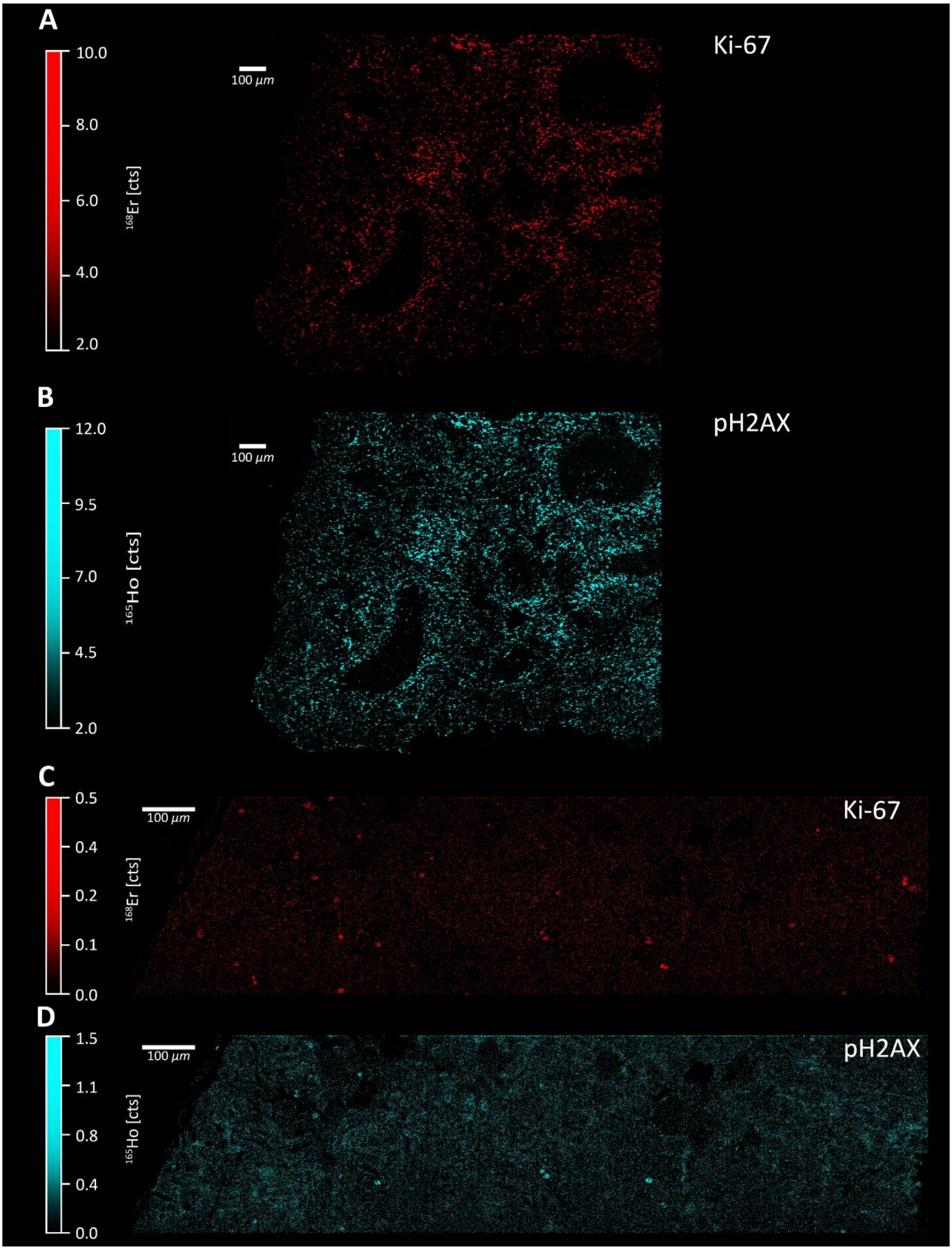

Figure S3: Cell proliferation and DNA damage in spleen and kidney tissue sections were visualized using the markers Ki-67 and pH2AX. LA-ICP-TOFMS data were acquired at a repetition rate of 500 Hz. The spleen images were recorded with a pixel size of 1 µm, and the kidney images with a pixel size of 500 nm. Spleen: (A) Ki-67, (B) pH2AX; Kidney: (C) Ki-67, (D) pH2AX.

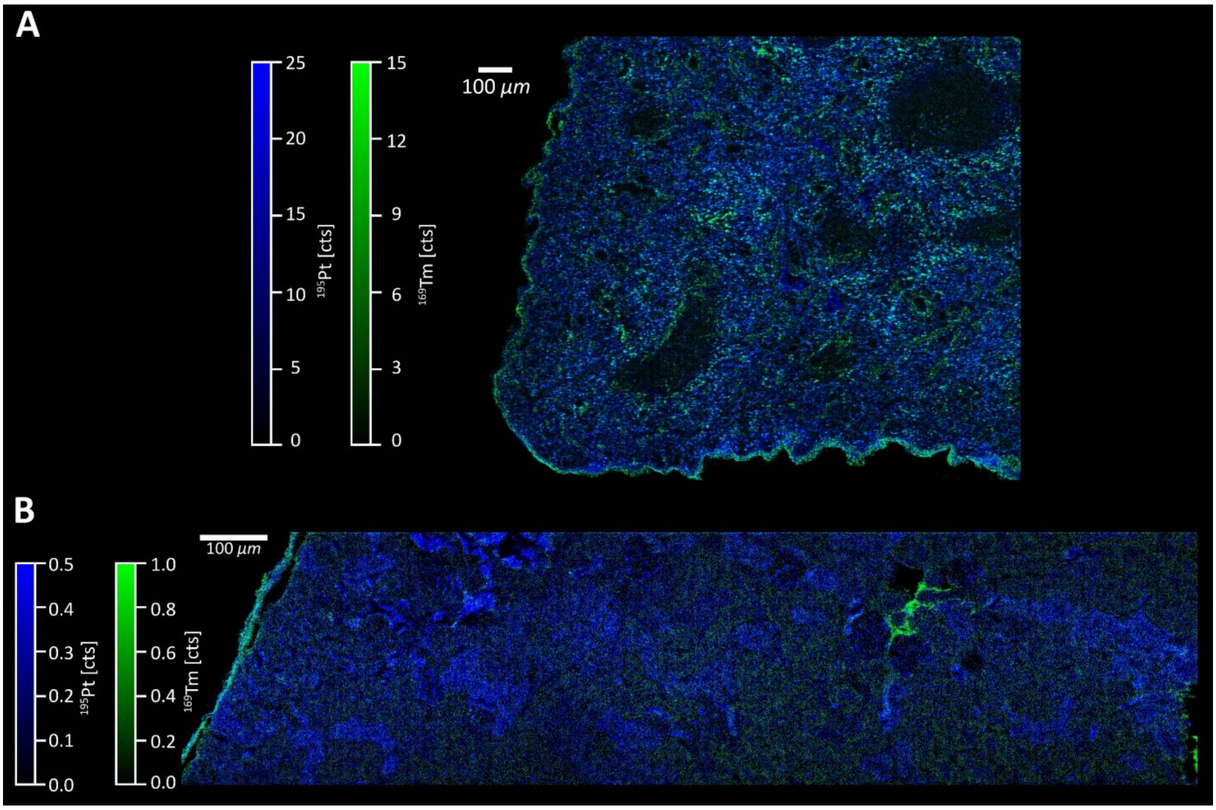

Figure S4: Overlay of platinum (^{195}Pt) and Collagen Type I (^{169}Tm) signals. LA-ICP-TOFMS data were acquired at a repetition rate of 500 Hz. The spleen images were recorded with a pixel size of 1 µm, and the kidney images with a pixel size of 500 nm. (A) Spleen; (B) Kidney.

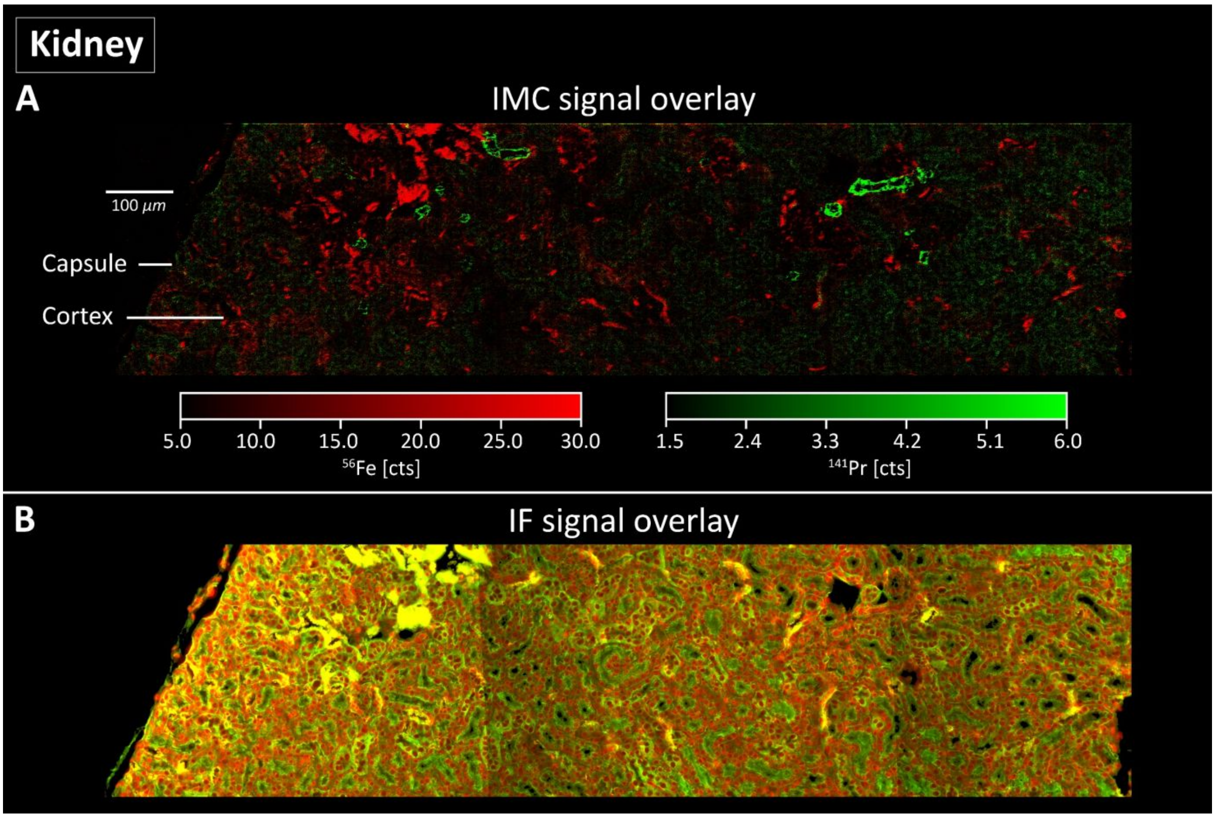

Figure S5: (A) shows an IMC signal overlay of iron (^{56}Fe) and α-SMA (^{141}Pr) in a kidney tissue section. The images were acquired at a pixel size of 500 nm and a repetition rate of 500 Hz, revealing distinct structural features in the kidney from an OxPt-treated mouse. The fluorescence overlay of DAPI and WGA in (B) provides an overview of the cellular structure.

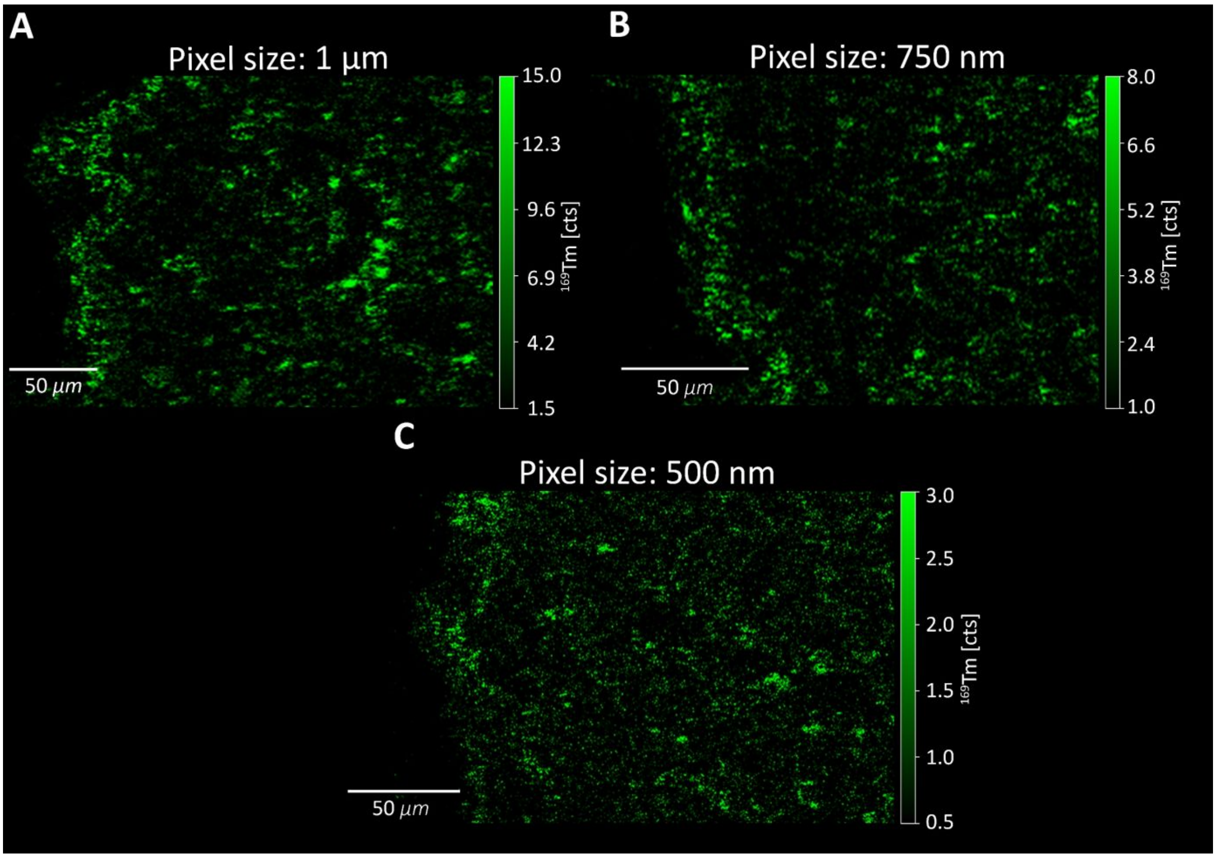

Figure S6: Collagen Type I intensity maps of a spleen tissue section. LA-ICP-TOFMS measurements were performed at a repetition rate of 500 Hz with different pixel sizes: (A) 1 µm, (B) 750 nm, and (C) 500 nm.

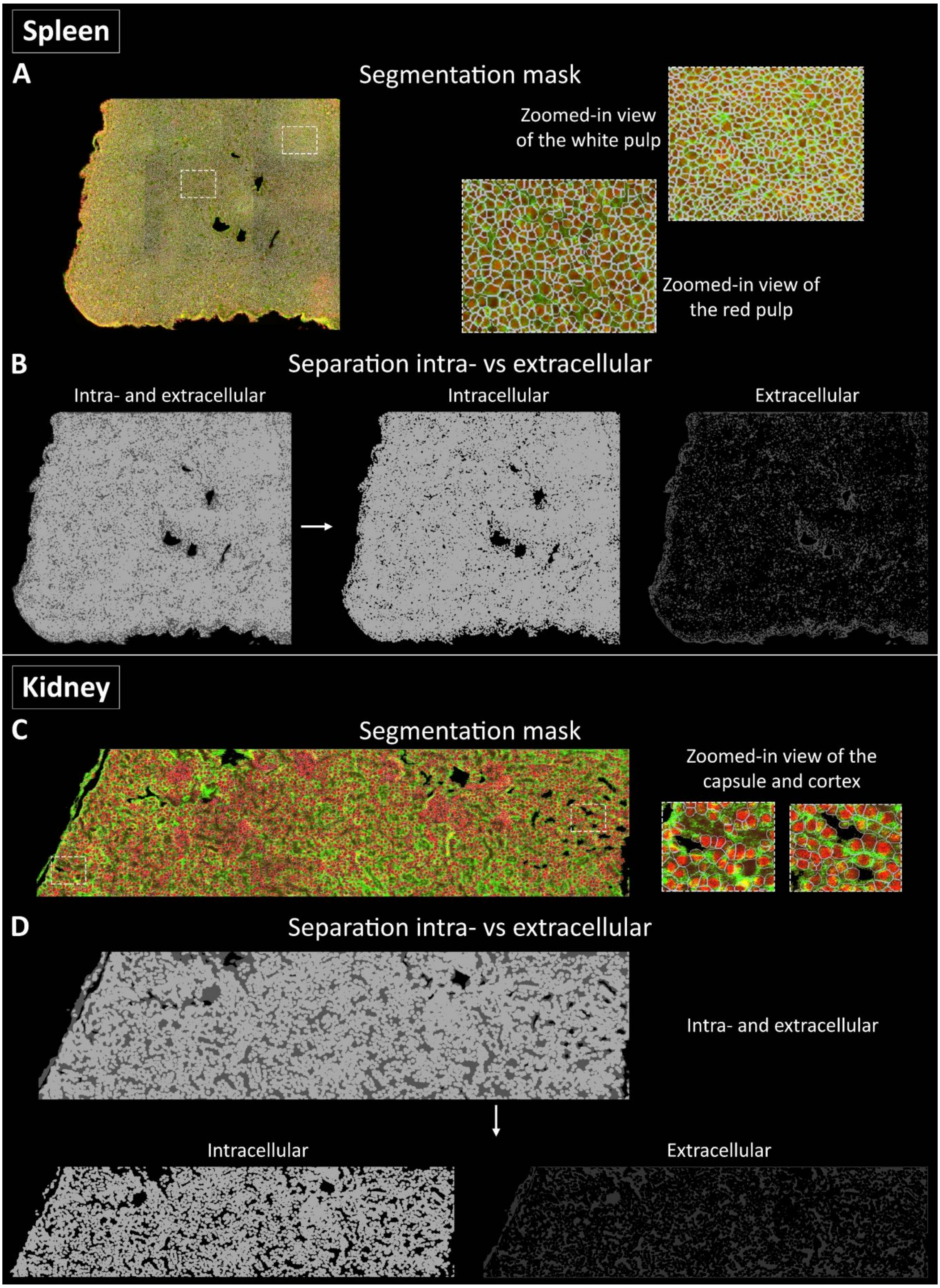

Figure S7: Segmentation and compartment validation. (A, C) Representative segmentation masks for spleen (based on DAPI-stained nuclei and wheat germ agglutinin (WGA)) and kidney (based on an iridium-based DNA intercalator and metal-labelled anti-WGA antibody (^{154}Sm)) sections, including zoomed-in views highlighting cell boundary delineation. (B, D) Visualization of the resulting intracellular masks and the corresponding non-cell-associated (extracellular) regions, defined as the complementary area not assigned to segmented cellular structures, which serve as the basis for spatially resolved quantification of Pt distributions.

Platinum quantification was performed at the pixel level using gelatine micro-droplet standards. Figure S7 illustrates the spatial distribution of the platinum signal across the spleen and kidney sections, where counts were converted into absolute amounts (femtograms) via external calibration.

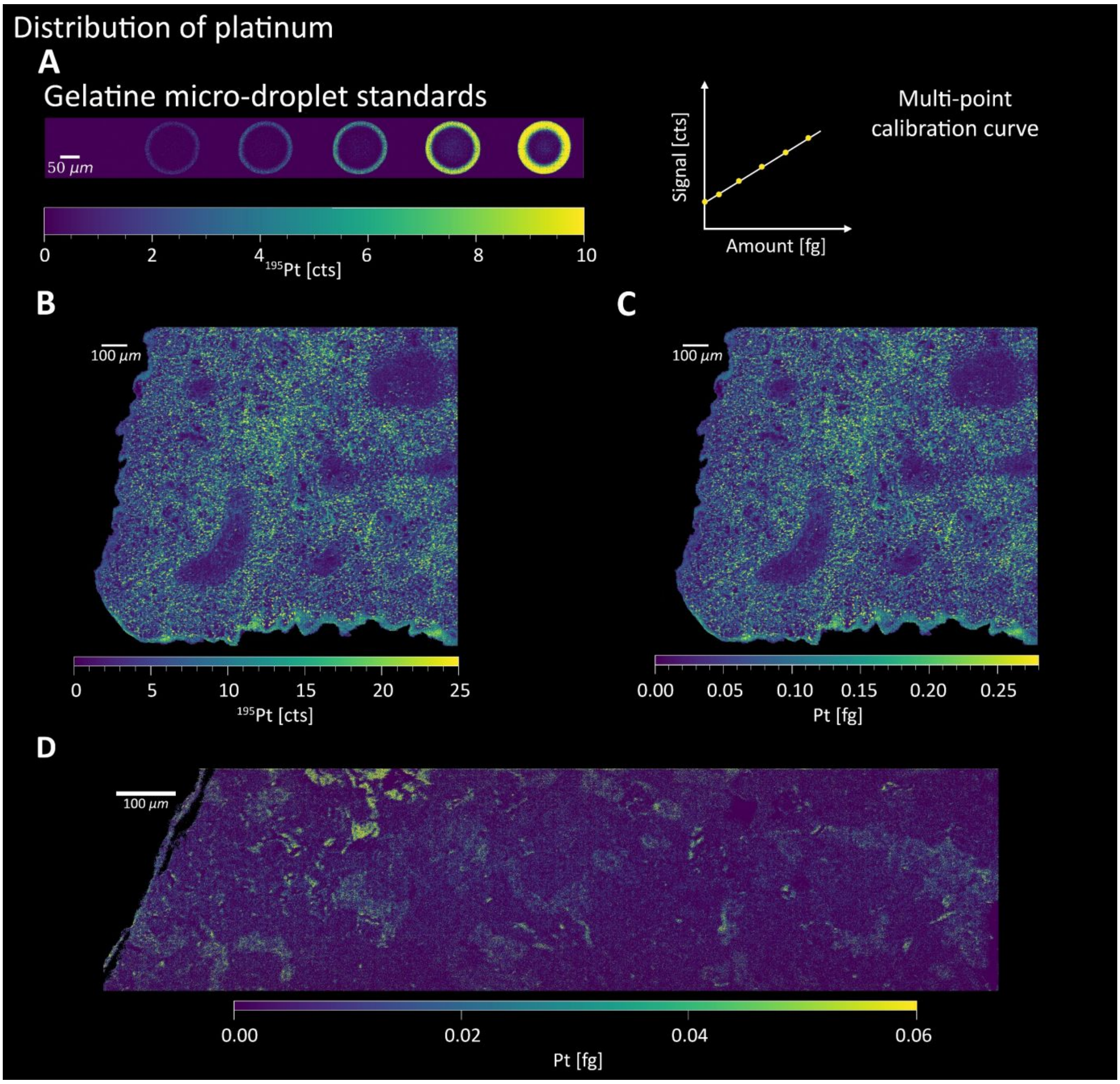

Figure S8: (A) illustrates a quantification series of gelatine micro-droplet standards, resulting in a multi-point calibration series, as shown in the schematic illustration. External calibration was used to convert platinum counts into absolute amounts. The absolute amount, given in femtograms, was obtained by dividing the counts by the slope of the external calibration curve. In (B), the platinum distribution in a spleen tissue section, acquired at a pixel size of 1 µm using LA-ICP-TOFMS, is shown in counts, and in (C), the corresponding platinum levels are converted into absolute amounts. In (D), the quantified platinum distribution of a kidney tissue section, acquired at a pixel size of 500 nm is shown.

Table S6: Quantitative analysis of selected regions of interest (ROIs) in the white pulp of a spleen tissue section. Data was acquired at a pixel size of 1 µm using LA-ICP-TOFMS. The table summarizes selected ROIs, distinguishing between intra- and extracellular compartments. Parameters include area [µm²] and absolute Pt mass [fg]. To account for the size differences between compartments, the area-normalized Pt content [fg/µm²] was calculated. Ratios (intra/extra or extra/intra) are provided to illustrate the Pt distribution. The overall cell density [cells/µm²] is reported relative to the total ROI area, enabling comparison between different ROIs.

	**Parameter**	**Intracellular**	**Extracellular**	**Ratio (Intra/Extra)**	**Ratio (Extra/Intra)**
White pulp 1					
**Measurements**	**Area [µm²]**	60271	2254	26.74	0.04
	**Amount [fg]**	1876	94	19.85	0.05
**Normalization**	**Areal density [fg/µm²]**	0.031	0.042	0.74	1.35
**Cell Statistics**	**Number of cells**	1188			
	**Cell density [cells/µm²]**	0.019			
White pulp 2					
**Measurements**	**Area [µm²]**	8918	1016	8.78	0.11
	**Amount [fg]**	321	56	5.70	0.18
**Normalization**	**Areal density [fg/µm²]**	0.036	0.055	0.65	1.54
**Cell Statistics**	**Number of cells**	161			
	**Cell density [cells/µm²]**	0.016			
White pulp 3					
**Measurements**	**Area [µm²]**	50538	3523	14.35	0.07
	**Amount [fg]**	1945	167	11.63	0.09
**Normalization**	**Areal density [fg/µm²]**	0.038	0.047	0.81	1.23
**Cell Statistics**	**Number of cells**	829			
	**Cell density [cells/µm²]**	0.015			

Table S7: Quantitative analysis of selected regions of interest (ROIs) in the red pulp of a spleen tissue section. Data was acquired at a pixel size of 1 µm using LA-ICP-TOFMS. The table summarizes selected ROIs, distinguishing between intra- and extracellular compartments. Parameters include area [µm²] and absolute Pt mass [fg]. To account for the size differences between compartments, the area-normalized Pt content [fg/µm²] was calculated. Ratios (intra/extra or extra/intra) are provided to illustrate the Pt distribution. The overall cell density [cells/µm²] is reported relative to the total ROI area, enabling comparison between different ROIs.

	Parameter	Intracellular	Extracellular	Ratio (Intra/Extra)	Ratio (Extra/Intra)
Red pulp 1					
**Measurements**	**Area [µm²]**	74684	9542	7.83	0.13
	**Amount [fg]**	7648	1122	6.82	0.15
**Normalization**	**Areal density [fg/µm²]**	0.102	0.118	0.87	1.15
**Cell Statistics**	**Number of cells**	1053			
	**Cell density [cells/µm²]**	0.013			
Red pulp 2					
**Measurements**	**Area [µm²]**	27040	2556	10.58	0.09
	**Amount [fg]**	2295	269	8.55	0.12
**Normalization**	**Areal density [fg/µm²]**	0.085	0.105	0.81	1.24
**Cell Statistics**	**Number of cells**	425			
	**Cell density [cells/µm²]**	0.014			
Red pulp 3					
**Measurements**	**Area [µm²]**	38086	4058	9.39	0.11
	**Amount [fg]**	2910	320	9.10	0.11
**Normalization**	**Areal density [fg/µm²]**	0.076	0.079	0.97	1.03
**Cell Statistics**	**Number of cells**	635			
	**Cell density [cells/µm²]**	0.015			
